# Teachers “Finding Peace in a Frantic World”: An Experimental Study of Self-Taught and Instructor-Led Mindfulness Program Formats on Acceptability, Effectiveness, and Mechanisms

**DOI:** 10.1037/edu0000542

**Published:** 2021-10-18

**Authors:** Jesus Montero-Marin, Laura Taylor, Catherine Crane, Mark T. Greenberg, Tamsin J. Ford, J. Mark G. Williams, Javier García-Campayo, Anna Sonley, Liz Lord, Tim Dalgleish, Sarah-Jayne Blakemore, MYRIAD team, Willem Kuyken

**Affiliations:** 1 Department of Psychiatry, University of Oxford; 2 Prevention Research Center, Pennsylvania State University; 3 Department of Psychiatry, University of Cambridge; 4 Department of Psychiatry, University of Zaragoza; 5 Medical Research Council Cognition and Brain Sciences Unit, University of Cambridge; 6 Cambridgeshire and Peterborough NHS Foundation Trust, Cambridge, United Kingdom; 7 Department of Psychology, University of Cambridge; 8 UCL Institute of Cognitive Neuroscience, London, United Kingdom

**Keywords:** teachers, well-being, mindfulness, self-compassion, mediation

## Abstract

Mindfulness training (MT) is considered appropriate for school teachers and enhances well-being. Most research has investigated the efficacy of instructor-led MT. However, little is known about the benefits of using self-taught formats, nor what the key mechanisms of change are that contribute to enhanced teacher well-being. This study compared instructor-led and self-taught MT based on a book (Williams & Penman, 2011) in a sample of secondary school teachers. We assessed expectancy, the degree to which participants believed the intervention was effective, their program engagement, well-being and psychological distress, and evaluated whether mindfulness and self-compassion skills acted as mediators of outcomes. In total, 206 teachers from 43 schools were randomized by school to an instructor-led or self-taught course—77% female, mean age 39 years (*SD* = 9.0). Both MT formats showed similar rates of participant expectancy and engagement, but the instructor-led arm was perceived as more credible. Using linear mixed-effects models, we found the self-taught arm showed significant pre-post improvements in self-compassion and well-being, while the instructor-led arm showed such improvements in mindfulness, self-compassion, well-being, perceived stress, anxiety, depression, and burnout. Changes over time significantly differed between the groups in all these outcomes, favoring the instructor-led arm. The instructor-led arm, compared with the self-taught, indirectly improved teacher outcomes by enhancing mindfulness and self-compassion as mediating factors. Mindfulness practice frequency had indirect effects on teacher outcomes through mindfulness in both self-taught and instructor-led arms. Our results suggest both formats are considered reasonable, but the instructor-led is more effective than the self-taught. Trial registration: ISRCTN18013311.

## Educational Impact and Implications Statement

Mindfulness training enhances teachers’ well-being. This study compared two different mindfulness training formats with a sample of 206 secondary school teachers using an experimental design. Results suggested that teachers’ well-being was increased in both formats of delivery and both generated similar expectancy and engagement with the mindfulness practice; but the instructor-led format appeared to be more credible and effective and was also able to reduce teachers’ stress, anxiety, depressive symptomatology, and burnout. In contrast, the self-taught program might be useful for those teachers who cannot access or commit to an instructor-led program, provided they engage with mindfulness practice. This study suggests two forms for teachers to learn mindfulness, instructor-led and self-taught, as a way to support well-being, each with its own advantages.

The majority of teachers enter the profession because they want to make a positive difference to young people’s lives (OECD, 2009), and for many people teaching is a valued vocation. However, teaching is a challenging profession that can involve significant work-related distress (Arvidsson et al., 2016). Maximizing teachers’ effectiveness and well-being, as well as minimizing occupational distress and turn-over, has been the subject of significant enquiry (Hakanen et al., 2006; Unterbrink et al., 2007; Van Horn et al., 1997; Wang et al., 2015). It has been observed that secondary school teachers show lower levels of well-being than is typical for the working-age general population (Kidger, Stone, et al., 2016). Poor well-being has implications for the health of the teachers and educational attainment of the children (Katz et al., 2018; Oberle & Schonert-Reichl, 2016), and the high levels of turnover and sickness absence create a financial burden on schools and on society more broadly (Naghieh et al., 2015). Our study is focused on how different models of mindfulness training (MT) might best support teachers to manage the demands of their teaching role in terms of perceived stress, as well as increase well-being, and reduce symptoms of anxiety, depression, and burnout. This study is part of a larger piece of work examining the efficacy, cost-effectiveness, mechanisms, and implementation of MT in schools (Kuyken et al., 2017; Montero-Marin et al., 2021). It also builds on two studies exploring different models of MT based on four training routes for school teachers wishing to deliver MT at school but differing in intensity and potential scalability (Crane et al., 2020), as well as the facilitators and barriers to implementing MT in schools (Wilde et al., 2019).

## Instructor-Led and Self-Taught MT for Enhancing Teacher Well-Being

There is a growing body of research investigating whether or not MT might be helpful for teachers in school settings. A recent meta-analysis of 29 studies of MT for teachers (including 1,493 participants between prekindergarten and 12th grade) suggests that MT has a medium effect size on a range of well-being and mental health outcomes (Klingbeil & Renshaw, 2018). Mindfulness is a natural, trainable, human capacity to bring awareness to all aspects of experience, with attitudes of curiosity, friendliness, and care (Bishop et al., 2004; Feldman & Kuyken, 2019). MT develops these foundational skills and enables people to apply them in their daily lives to support well-being and general functioning. MT combines regular mindfulness exercises with psycho-educational content, designed to provide a rationale for the program, and an enhanced understanding of psychological processes that are relevant to the specific population receiving the program (Crane et al., 2017; Jennings & DeMauro, 2017). The practice of mindfulness meditation is a core component of MT that seems to be related to mental health outcomes such as decreased rumination, depressive symptom alleviation, and lower hazard of relapse to major depression (Crain et al., 2017; Crane et al., 2014; Hawley et al., 2014; Segal et al., 2013). The meta-analysis carried out by Parsons et al. (2017) observed small but significant associations between the amount of mindfulness practice and reductions on stress, anxiety and depressive symptomatology.

A commonly used format for MT for teachers is an instructor-led eight-session group delivery (Lomas et al., 2017), possibly derived from the original structure of major mindfulness-based programs, such as mindfulness-based stress reduction and mindfulness-based cognitive therapy. Over the course of the program, participating teachers engage in a range of mindfulness practices and psycho-educational exercises. These are intended to develop their ability to attend to present moment experiences in a nonreactive and nonjudgmental way in order to become aware of, and relate differently to, unhelpful mental habits (Crain et al., 2017). Mindfulness instructors encourage and support participants’ to engage in mindfulness practices that can translate to their everyday lives, both professionally and personally. However, a major barrier for many people is accessing instructor-led MT programs. Among school teachers, lack of time and financial resources have been identified as the most important barriers (Bazzano et al., 2018; Wilde et al., 2019).

An important challenge for the field is therefore finding accessible and scalable ways for people to learn how to use MT to support their well-being (Lim et al., 2015). In this sense, mobile apps, web-based programs, and bibliotherapy are potential ways to enhance access to MT (Cavanagh et al., 2014). Preliminary findings suggest that these formats might have some benefits for well-being in college students and people with a history of depression (Hazlett-Stevens & Oren, 2017; Lever-Taylor et al., 2014; Levin et al., 2020; Segal et al., 2020). Such self-taught MTs could increase accessibility for teachers, enabling them to choose when and how to engage with mindfulness practice. However, greater accessibility can come at the cost of lower engagement, which is a relevant consideration given that research suggests that engagement with mindfulness practice is a key element of possibility for change (Roeser et al., 2013; Webb et al., 2017).

The mindfulness training manual, *Mindfulness: A Practical Guide to Finding Peace in a Frantic World* (M-FP; Williams & Penman, 2011), was first written as a “self-guided” book intended to be a highly accessible, low-intensity introduction to mindfulness meditation practice suitable for the general population. Using a sample of undergraduate students versus waitlist controls, a study by Lever-Taylor et al. (2014) suggested that the M-FP self-taught program was associated with large effect sizes for mindfulness and moderate effect sizes for self-compassion as well as perceived stress, depression, and anxiety. An instructor-supported program based on the book has also been developed and is being taught widely, including to school teachers. A previous controlled study of secondary school teachers used a very similar instructor-supported group program led by qualified mindfulness instructors and observed large effect sizes for mindfulness, self-compassion, well-being, and perceived stress (Beshai et al., 2016). However, we do not know the comparable effectiveness of self-taught and instructor-led formats of teacher MT.

## Acceptability and Engagement With Self-Taught and Instructor Led MT

Research is needed to compare self-taught and instructor-led methods of MT delivery with regard to not just effectiveness (e.g., impact on mechanisms and outcomes) but also to implementation factors such as acceptability and engagement with the MT, which could facilitate teacher skills development (Roeser et al., 2012). Acceptability is the degree to which individuals perceive an intervention as reasonable and appropriate to their needs, and this can be based on anticipated responses to the MT, for example, expectancy for positive outcomes previous to having experiences with the program, or based on experienced responses, for example, credibility of the program after having completed it (Sekhon et al., 2017). Acceptability is considered essential for influencing usage and results of a given intervention, because programs that are expected to be acceptable are more likely to be used with a greater degree of integrity (Witt & Elliott, 1984). In this sense, engagement with meditation exercises, for example, frequency of mindfulness meditation practice, is a core integrity aspect of mindfulness-based programs, as adherence to meditation practice underlies its theoretical model of psychological change (Crane et al., 2017). Other important characteristic of engagement is the dose or amount of program that participants receive (Durlak & DuPre, 2008). In the case of instructor-led MT, dose can be operationalized as the number of group sessions that participants receive. However, in the case of self-taught MT, we need to consider the specific format through which the MT was accessed (e.g., if the MT was in the form of a workbook, such as the M-FP program, how much of the book the participants read is the dose variable).

In summary, we need to know more about the comparable effectiveness of self-taught and instructor-led formats of teacher MT, but also to explore possible differences in acceptability (e.g., expectancy for positive outcomes, and credibility after carrying out the program), and engagement (e.g., frequency of mindfulness meditation practice, and amount of the program that has been received). In this study, we examine possible differences in those perceptions, experiences, and outcomes related to each type of MT delivery in teachers. We would expect more positive values in the instructor-led MT, as it has the support of an expert who drives the motivation of participants toward the aims of the program.

## Mindfulness and Self-Compassion Skills as Psychological Mechanisms of MT

If an adequate MT implementation brings about positive change in well-being and mental health for secondary school teachers, this raises the question of how and why these effects are produced and what the importance of such changes are for a teachers’ professional role. A comprehensive theoretical framework hypothesizes that an adequate implementation of MT could enable teachers to develop skills such as mindfulness and self-compassion, which if applied in daily life through practice, lead teachers to improved occupational health and well-being (Roeser et al., 2012; Roeser et al., 2013). Specifically, MT seems to promote mindfulness and self-compassion by improving the ability to intentionally focus attention on the here and now, instead of ruminating about the past or worrying about the future (Kabat-Zinn, 1994), and also, cultivating a certain attitude of curiosity toward the present moment that suspends self-criticism and facilitates coping with challenges with kind acceptance (Cullen, 2011). All of this in turn would reinforce teachers’ resilience, favoring adequate coping processes (Taylor et al., 2016), and supporting a “prosocial classroom” that starts with the teachers’ well-being (Jennings & Greenberg, 2009).

There is growing evidence that enhancements in mindfulness and self-compassion skills could play an important role in promoting mental health and well-being (Gu et al., 2015; Van der Velden et al., 2015). Self-compassion is defined as “being touched by and open to one’s own suffering, not avoiding or disconnecting from it, generating the desire to alleviate one’s suffering and to heal oneself with kindness” (Neff, 2003). In general, mindfulness skills enable people to recognize their distress without judgment, be open to it, and learn to self-soothe through a range of strategies, whereas self-compassion skills might specifically increase the ability to regulate intense negative emotions in response to stressors (Emerson et al., 2017; Hölzel et al., 2011; Kirschner et al., 2019; Neff & Dahm, 2017).

These skills are helpful to teachers because teaching can be a stressful occupation, and teachers need abilities to manage their own negative emotional responses (Carson et al., 2006). In fact, failure to do so can lead to a “burnout cascade,” impairing physical and mental health and producing anxiety and depressive symptoms (McEwen, 2008). This is particularly problematic in a classroom context because increased teacher irritability and distress has the potential to produce a similar reactivity in the pupils (Jennings & Greenberg, 2009). A previous study observed that teachers randomized to instructor-led MT showed greater mindfulness, self-compassion, focused attention, and working memory as well as lower levels of stress and burnout than waitlist controls; with mindfulness and self-compassion skills mediating reductions in stress, burnout, anxiety, and depression (Roeser et al., 2013). Thus, these foundational skills of mindfulness and self-compassion may increase teachers’ ability to cope with their everyday job and could be thus relevant across the spectrum of wellness in teachers, with the potential to move teachers toward improved states of mental health and well-being (Crain et al., 2017; Klingbeil & Renshaw, 2018; Roeser et al., 2013; Schussler et al., 2018; Schussler et al., 2019; Taylor et al., 2016).

It has also been observed that mindfulness meditation practice facilitates staying well after depression (Crane et al., 2014), and that indirect effects on depressive symptoms may occur through reductions in rumination (Hawley et al., 2014). MT might facilitate the identification of ruminative patterns, understanding the consequences of these kinds of thoughts and taking constructive steps to reduce mental distress (Smart et al., 2016). Exploratory studies have observed that MT aimed at reducing ruminative thinking in depressive patients also improves mediators such as mindfulness and self-compassion, although more research to establish the specific causal links between these two groups of variables is needed (Foroughi et al., 2020; Frostadottir & Dorjee, 2019). These indirect effects of mindfulness practice have been observed in clinical samples using instructor-led MT, but they have not been studied in secondary teachers using self-taught MT. Therefore, we do not know the relative importance of the mindfulness practice when using these different delivery formats. We also do not know whether mindfulness and self-compassion might differently mediate gains in outcomes according to these two modes of delivery.

We would expect different patterns of mediation because those receiving the instructor-led course are potentially exposed to greater modeling of mindfulness and self-compassion by the class instructor as he or she responds to participants’ sharing of personal experiences. The fact that the class participants are all listening to and learning from each other’s experiences, and at the same time are supported and guided by an expert who holds and befriends the group, might enhance opportunities to learn mindfulness and self-compassion skills, increase participants’ sense of common humanity, and also reduce stigma associated with mental health difficulties (Allen et al., 2009; Griffith et al., 2019; Tickell et al., 2020). Thus, we proposed that the mindfulness and self-compassion skills might be acquired and mediate outcomes in different ways across the two MT formats.

## Study Aims

The first aim of this study was to explore possible differences in the levels of acceptability (e.g., expectancy for positive outcomes, and credibility of program content after completing the program) and engagement with the program (e.g., frequency of mindfulness meditation practice, and amount the book read) of two MT delivery formats (e.g., self-taught and instructor-led) of the popular mainstream manual, M-FP (Williams & Penman, 2011), as well as their effectiveness on the well-being and mental health of secondary school teachers. The second aim was to explore possible mechanisms of change, specifically whether foundational skills such as mindfulness and self-compassion mediate improvements in well-being and mental health in secondary teachers, and whether frequency of mindfulness practice might produce indirect effects on outcomes, using self-taught and instructor-led ways of delivering the MT (we only examined frequency of practice because we do not have enough evidence to consider other implementation variables as potentially amplifying indirect effects in MT). We recognize that a third arm receiving no intervention (which would allow us to know the usual course of development across the time) would have strengthened the study design used. Nonetheless, our study enables us to answer important questions about the expectancy, credibility, engagement, effectiveness, and mechanisms of change of two modes of MT in secondary school teachers, and we are able to benchmark our findings in terms of mental health, perceived stress, anxiety, depression, and burnout, as well as mindfulness and self-compassion, using previous studies which have compared teacher MT to no intervention control groups (Beshai et al., 2016; Lever-Taylor et al., 2014; Roeser et al., 2013).

## Method

The study was registered at the ISRCTN trials registry on November 24, 2015, prior to obtaining participant consent to randomization (ISRCTN18013311). The data reported in this article form part of a larger experimental study using a four-arm cluster randomized feasibility design to examine the implementation processes and competency reached by different training pathways for secondary school teachers wishing to deliver a MT program to their students (Crane et al., 2020) English secondary schools (clusters) were randomized and outcomes were measured on participating teachers within these schools. The first phase in training to deliver MT to students was to follow a personal MT program that was intended to support participant teachers learning mindfulness for their own well-being. The aim was to encourage participant teachers to have experience with, and an understanding of, mindfulness (Phase 1) prior to teaching it to students (Phase 2). The data reported here correspond to the first phase of the protocol and indicate prepost changes of MT on the participants’ psychological mental health and well-being via self-taught and instructor-led formats of the M-FP program.

### Recruitment

We recruited secondary schools that were representative of mainstream English schools in terms of the proportion that were state funded, large, selective, and with above average levels of pupil deprivation (e.g., percentage of pupils that were receiving free school meals). The characteristics of participating teachers in relation to the national age and gender of the secondary school teacher workforce were also monitored. Recruitment was conducted through emails sent directly to all secondary school head-teachers and local education authorities in England, identified through a freedom of information request. Potential participant teachers and headteachers were also approached through professional events (such as local headteacher meetings). Interested individuals (whether headteachers or teaching staff) were invited to contact the research team. Initially, contacts were received from 185 schools, and following screening, this resulted in 254 eligible and interested teachers from 75 schools. After exclusion of schools with fewer than three participating members of staff and schools located in a geographical region too logistically difficult to be reached by a mindfulness instructor, 206 participating teachers from 43 schools were ultimately included in the study. Schools were then randomized. School and participating teacher inclusion/exclusion criteria are reported in the Supplementary Material S1. The baseline characteristics of those schools and participating teachers who started the study are shown in the Supplementary Material S2.

### Procedure

Following receipt of the study information, interested teachers were screened for eligibility and provided written informed consent. In order for a participating teacher to be included in the study, consent was initially required from the head-teacher of the school. Consenting participating teachers were sent a link to an online questionnaire containing the baseline measures, which were completed in Autumn 2015. Once at least three participating teachers within each school had completed this baseline preintervention assessment (T0), the school was then eligible to be included in both Phase 1 (teacher mindfulness) and Phase 2 (teacher training for student mindfulness program), being randomized to one of four training routes. Two of the training routes commenced with self-taught MT (Phase 1: self-taught teacher mindfulness + Phase 2: 1-day syllabus training for student mindfulness program; and Phase 1: self-taught teacher mindfulness + Phase 2: 4-day syllabus training for student mindfulness program), and two commenced with instructor-led MT (Phase 1: instructor-led teacher mindfulness + Phase 2: 1-day syllabus training for student mindfulness program; and Phase 1: instructor-led teacher mindfulness + Phase 2: 4-day syllabus training for student mindfulness program). Schools were randomized using a simple randomization procedure, with equal probability of allocation for each cluster (school) within strata (schools that recruited five or more teachers or less than five teachers). We anticipated that having more teachers in a school might influence training through greater opportunities for peer learning, support, and positive group experience, and therefore, we stratified the randomization of schools (clusters) based on the number of participating teachers per school. The cut was the median. Randomization was conducted by Exeter Clinical Trials Unit (ExeCTU), which was not otherwise involved in the study. MT commenced in February 2016 and was delivered free of charge. Following completion of the instructor-led course, or the end of a comparable time frame allotted to completion of the self-taught course, participating teachers were sent a second link to complete the postintervention assessment (T1). Participating teachers were compensated £25 in Amazon vouchers for completion of each set of study questionnaires. Schools were also given £250 to spend on school resources at the end of the study. Completion of the post intervention (T1) assessment marked the end of study Phase 1, which corresponded to the personal-training intended to support participating teachers learning mindfulness skills for themselves. Participating teachers then continued to Phase 2, which focused on the training required to deliver a MT curriculum to pupils. The corresponding second phase training route was not communicated until after they had commenced Phase 1 training (details of Phase 2 training routes and outcomes can be seen in Crane et al., 2020).

### Mindfulness Trainings

The two different forms of MT examined in this study were both based on the M-FP manualized curriculum (Williams & Penman, 2011). This curriculum is an introductory skills-based training designed to be applicable for use in everyday life. It builds on the core elements of mindfulness-based cognitive therapy (Segal et al., 2013) and was developed specifically for improving well-being in the general population. It develops the following understandings and skills: (a) recognizing the tendency to be on autopilot and begin to practice bringing mindfulness to aspects of everyday present-moment experience; (b) recognizing thoughts, emotions, sensations, and impulses, stabilizing attention and returning with appreciation to the here and now; (c) recognizing unhelpful patterns of thinking, feeling, and acting; and (d) learning skills for keeping balanced through life’s ups and downs, responding skillfully when difficulties arise, engaging with what is most important, and opening up to moments of joy, contentment, and gratitude. The curriculum has eight sessions that work sequentially through these four main themes. Participants are encouraged to engage in a range of home practices that support learning, including a daily 20-minute mindfulness practices.

#### Self-Taught Mindfulness Training

Participating teachers allocated to self-taught training were provided with the M-FP course book (Williams & Penman, 2011). Each participating teacher was contacted prior to commencing the course and the importance of reading the whole course book and completing the associated activities and mindfulness practices was emphasized. Participating teachers were asked to read the introductory chapters of the M-FP book to ensure that a minimum preliminary knowledge had been reached before they were able to commence the 8-week program outlined in the course book on a set date (usually the week after mailing out the books and as far as possible contemporaneous with instructor-led MT groups). Participating teachers were also given access to a publicly-available app which accompanies the course and a CD or MP3 of the course material. A general overview and some details of the 8-week program timeline, themes, and specific mindfulness practices, so that one can see the distribution and nature of the contents and activities carried out and how they train mindfulness and self-compassion through meditation exercises, is included in the Supplementary Material S3.

#### Instructor-Led Mindfulness Training

The instructor-led delivery was based on the same M-FP manual (Williams & Penman, 2011), and was taught face-to-face (in person) in groups of between three and nine participating secondary teachers at their corresponding school facilities. Participating teachers also read the book by Williams and Penman (2011) alongside their group sessions.

The course was delivered by trained and experienced mindfulness instructors over eight 90-min group sessions, occurring once per week. We trained 47 qualified mindfulness instructors that (a) had taught at least five classes since their MT qualification (preferably mindfulness-based cognitive therapy); (b) had a desire to work with secondary school teachers in a school setting; and (c) were registered on the United Kingdom listing of mindfulness trainers and adhered to the Good Practice guidelines for mindfulness instructors (Supplementary Material S4). A 2-day training course was provided to the pool of 47 potential trainers, which consisted of presenting project and school information and going through the M-FP teacher handbook with experienced supervisors and program developers. A general overview of the research project and the course was given and allowed the trainers to become familiar with all the materials and practices to be used. In the end, 15 mindfulness instructors that agreed with the responsibilities of the study (Supplementary Material S4) were chosen following a process of matching the location and dates for each school with the nearest mindfulness instructor (once the schools had been randomized then the trainers were matched, first by geographic location, i.e., nearest to the school, and then on availability on the days that the school had specified). Biweekly supervisions, regular telephone support and emails throughout the course were conducted with experienced supervisors in the M-FP course, with mindfulness instructors encouraged to get in touch if they need any kind of help.

Mindfulness instructors were asked to record one session of their classes to bring it to their experienced supervisor for discussion and to ensure the quality of the course and standardization control. Members of the research team were in contact with the mindfulness instructors, introducing them to schools and providing a box which contained everything they would need practically for the course. If a mindfulness instructor was involved, they were paid £1,300. An overview of the course is shown in the Supplementary Material S3.

### Measurements

As observed in Table 1, we collected data on the following school level sociodemographic characteristics: percentage of pupils eligible for free school meals (i.e., school level of pupil deprivation), school funding status (state schools or independent), school size (big ≥ 1,000 pupils, small < 1,000 pupils), school quality rating (Office for Standards in Education rating, OFSTED) of state schools, and number of participating teachers in school. We also collected data on participating teachers’ age, gender, marital status, and number of years teaching. To measure participating teacher views of implementation quality of the program they took, we measured expectancy for positive outcomes and credibility of program material, as well as teachers' engagement with the program. We also measured psychological mechanisms of change (e.g., mindfulness and self-compassion), and psychological well-being, distress and occupational health.[Table tbl1]

#### Expectancy and Credibility

We used five questions adapted from a previous school-based mindfulness study (Bluth et al., 2016) that measure the degree to which participants believe that the intervention is effective in improving outcomes. To measure expectancy, this scale was implemented at the second week of the intervention (T0b), ensuring that participants had an understanding of what the program would entail. It was also used immediately postintervention (T1), as a measure of perceived credibility of the program. This ad-hoc unidimensional scales use a Likert-type scale ranged between 0 (*not at all*) and 10 (*a great deal*), and include items related to the sense of the program (expectancy: “How much does what’s being taught in this course make sense to you?”; credibility: “How much did what was taught in the course make sense to you?”), expected improvements (expectancy: “How confident are you that this course will help improve your well-being?”; credibility: “How much do you believe that the course has improved your well-being?”), likelihood of recommending the program to a friend (this item is shared by both expectancy/credibility: “How confident would you be in recommending the course to a friend?”), importance of the program (this item is shared by both expectancy/credibility: “How important do you think it would be to make the course available to other teachers?”), and expected success (this item is shared by both expectancy/credibility: “How successful do you believe the course would be in decreasing problems or issues that teachers have?”). The one-factor solution structure showed adequate goodness-of-fit indices for both scales (Supplementary Material S5). The internal consistency obtained for expectancy (T0b) was ω = .90, and for credibility (T1) was ω = .92.

#### Engagement With the Program

Participants in both self-taught and instructor-led arms had to complete questions asking about their engagement with the program at the postintervention (T1) assessment. The number of days per week on which the participant had completed mindfulness meditation practices (frequency of practice) during the program was asked as follows: “During the period that you were following the course, on how many days per week, on average, did you complete at least one mindfulness practice (e.g., a guided meditation such as the body scan, breath and body, sounds and thoughts, mindful movement, etc.).” The response options for this question ranged between 0 and 7. In addition, a homework booklet was used to register the practices that were carried out throughout the program, and all participating teachers were asked to return their homework booklets. In total, 93 booklets were returned by the participating secondary teachers to the research group. There were no significant differences between groups in terms of returning booklets: the self-taught group returned 42 (42%) booklets, while the instructor-led group returned 49 (47%) booklets (χ^2^ = .54; *p* = .463). These booklets were only used to evaluate the accuracy of the self-reported data on the frequency of mindfulness practice at postintervention (T1), which was used in the subsequent analyses, yielding a convergence value between them of Spearman’s *ρ =* .65.

Participants in both self-taught and instructor-led arms also provided information on the number of chapters of the course book (i.e., reading the book) that they had read using the following scale: *none*, *just introductory chapters*, *less than four of the chapters outlining the 8-week course*, *more than four of the chapters outlining the 8-week course*, or *the whole book*. Finally, we recorded reasons for drop-out from the protocol as well as the number of group sessions attended by participants of the instructor-led group.

#### Psychological Mechanisms of Change

Mindfulness was measured using the Five Facet Mindfulness Questionnaire-Short Form (FFMQ-SF; Gu et al., 2016). The FFMQ-SF is a 15-item questionnaire which includes three items for each of the five mindfulness facets of observing (e.g., “I notice how foods and drinks affect my thoughts, bodily sensations, and emotions”), describing (e.g., “I’m good at finding words to describe my feelings”), acting with awareness (e.g., “I do not pay attention to what I’m doing because I’m daydreaming, worrying, or otherwise distracted”, item reversed), nonjudging of inner experience (e.g., “I tell myself I shouldn’t be feeling the way I’m feeling”, item reversed), and nonreactivity to own thoughts (e.g., “When I have distressing thoughts or images I am able just to notice them without reacting”). The items are rated on a 5-point Likert scale, ranging from 1 (*never true*) to 5 (*always true*), with higher scores indicating greater dispositional mindfulness (range: 15–75). Following the validation work of this questionnaire (Gu et al., 2016), a second-order factor model that allows the use of a single total score was evaluated showing adequate goodness-of-fit (Supplementary Material S5). Given that the inspection of mindfulness as a one-dimensional construct is a parsimonious and interpretable option consistent with previous research (e.g., Gu et al., 2016), we used a total score calculated by means of the sum of all the items—reversed when necessary—as a measure of trait mindfulness (T0 ω = .82, T1 ω = .87).

The Self-Compassion Scale-Short Form (SCS-SF) is a 12-item questionnaire (Raes et al., 2011) that assesses how respondents perceive their actions toward themselves at times of difficulty. Items are rated using a Likert-type scale, from 1 (*almost never*) to 5 (*almost always*). In the present study, we selected out the “mindfulness” items given their overlapping conceptual and operational definitions with the FFMQ-SF as a way to handle redundant item content across these two measures (Roeser et al., 2013). Thus, only the 10 items corresponding to self-kindness (e.g., “When I’m going through a very hard time, I give myself the caring and tenderness I need”), self-judgment (e.g., “I’m intolerant and impatient toward those aspects of my personality I do not like”), common humanity (e.g., “I try to see my failings as part of the human condition”), isolation (e.g., “When I fail at something that’s important to me, I tend to feel alone in my failure”), and overidentification (e.g., “When I fail at something important to me I become consumed by feelings of inadequacy”) were included. Using a bifactor model, a recent study has obtained evidence for the calculation of a total score by means of the sum of all the items after reversing the negative ones (Neff et al., 2019). In order to test the viability of this, we evaluated the bifactor model on the 10 items considered. Results showed adequate goodness-of-fit (Supplementary Material S5), allowing us the use of a parsimonious total score. Higher scores indicate greater self-compassion (mean scores were used, range: 1–5). The internal consistency was T0: ω = .90, T1: ω = .90.

#### Psychological Well-Being, Distress, and Occupational Health

The Warwick Edinburgh Mental Well-being Scale (WEMWBS) was developed as a unidimensional tool to evaluate mental well-being in the general population (Tennant et al., 2007). It is a 14-item scale (e.g., “I’ve been feeling optimistic about the future”; “I’ve been interested in new things”) with five Likert-type response categories from 1 (*none of the time*) to 5 (*all of the time*). Items are worded positively—and therefore higher scores indicate greater levels of mental well-being—and cover both feeling and functioning aspects of mental well-being (sum scores were used, range: 14–70). The one-factor structure of the WEMWBS had appropriate goodness-of-fit indices in our study (Supplementary Material S5). The internal consistency obtained was T0: ω = .91, T1: ω = .93.

The Perceived Stress Scale (PSS) is a self-report instrument that measures the degree to which different situations in one’s life are appraised as stressful (Cohen & Williamson, 1988). It includes 10 questions about feelings and thoughts during the last month (e.g., “How often have you felt that you were unable to control the important things in your life?”; “How often have you found that you could not cope with all the things that you had to do?”), that can be answered by a Likert-type scale from 0 (*never*) to 4 (*very often*). Higher scores reflect greater levels of perceived stress (sum scores were used, range: 0–40). The PSS has shown a unidimensional structure (Roberti et al., 2006) that was replicated in our study with appropriate goodness-of-fit indices (Supplementary Material S5). The internal consistency values of the PSS obtained in the present study were T0: ω = .90, T1: ω = .89.

The Patient Health Questionnaire-9 (PHQ-9) is a brief unidimensional self-report instrument that can monitor changes in the severity of depressive symptomatology in response to interventions (Kroenke et al., 2001). It consists of nine items that ask how often participants have been bothered over the past 2 weeks (e.g., “Little interest or pleasure in doing things”; “Feeling down, depressed, or hopeless”), and are scored using a Likert-type scale from 0 (*not at all*) to 3 (*nearly every day*). Higher scores represent a greater severity of depressive symptomatology (sum scores were used, range: 0–27). The one-factor structure obtained adequate goodness-of-fit indices in the present study (Supplementary Material S5), with internal consistency values of T0: ω = .80, T1: ω = .84.

The General Anxiety Disorder-7 (GAD-7) is a unidimensional questionnaire consisting of seven items to measure generalized anxiety symptoms (Spitzer et al., 2006). Each item ask how often participants have been bothered over the past 2 weeks (e.g., “Not being able to stop or control worrying”; “Feeling nervous, anxious, or on edge”) using a Likert-type scale from 0 (*not at all*) to 3 (*nearly every day*), with higher scores indicating greater severity of anxiety symptoms (sum scores were used, range: 0–21). This one-factor structure obtained adequate goodness-of-fit indices (Supplementary Material S5), with internal consistency values in the present study of T0: ω = .90 and T1: ω = .87.

The Maslach Burnout Inventory-Educators Survey (MBI-ES) is a 22-item questionnaire designed to assess burnout in teachers (Maslach et al., 1996) through the components of emotional exhaustion (e.g., “I feel emotionally drained from my work”), depersonalization (e.g., “I feel I treat some students as if they were impersonal objects”), and (lack of) personal accomplishment (e.g., “I deal very effectively with the problems of my students”; item reversed). Items ask about personal feelings or attitudes toward the teaching work and are rated by the frequency with which they are experienced on a Likert-type scale from 1 (*never*) to 7 (*every day*). Recent studies using this questionnaire have proposed the use of a single total score, and evidence of this has been observed in teachers through bifactor models (Szigeti et al., 2017). Following this suggestion, we tested the bifactor model obtaining adequate goodness-of-fit indices (Supplementary Material S5). This supports using a single total MBI-ES score, calculated as the sum of all the items after reversing the ones included in the personal accomplishment domain. Higher values indicate greater burnout symptomatology (range: 22–154). The MBI-ES internal consistency values obtained in the present study were T0: ω = .85, T1: ω = .87.

While we used scale totals for the analyses, we examined the correlations between subscales in those scales that are theoretically formed by subfactors, as well as the correlations between the total scores of all the scales, in order to oversee their degree of convergence (Supplementary Materials S6 and S7).

### Ethics

The study was approved by the University of Oxford Ethics Committee (20/03/2015; ref. MS-IDREC-C1-2015-048), and it was also overseen by a Data Monitoring Committee at each stage of the process. Mindfulness trainers did not report any contraindications with potential participants, and did not report any safeguarding issues concerning risk of harm. Participants reporting high scores on questionnaires (i.e., above their corresponding established cut-offs) were managed anonymously within the study risk and safeguarding protocol. All teachers were treated in accordance with the ethical standards of the American Psychological Association (APA).

### Data Analysis

#### Descriptive Data at Baseline

To check whether randomization had delivered balance across the groups, descriptive statistics (*means*, *SD*s, medians, interquartile ranges, frequencies, and percentages, according to the level of measurement and statistical distribution of each variable), and between-group comparisons by means of a chi-square (or Fisher’s when necessary), Mann-Whitney, or *t*-test were computed for an inspection of variables across arms at baseline.

#### Implementation Variables: Expectancy, Credibility, and Engagement With the Program

We also described and examined whether acceptability (e.g., anticipated responses of expectancy previous to having a total experience with the program; and experienced responses of credibility of the program after having completed it), as well as engagement with the program (e.g., frequency of weekly mindfulness meditation practice, and amount of reading the book), differed across groups by means of Mann-Whitney or *t*-test, depending on the level of measurement and statistical distribution of the corresponding variables. We also explored whether expectancy (i.e., a measure that was taken in the second week of the intervention) was a predictor of frequency of mindfulness practice, and amount that the book was read during the program (and additionally, the number of group sessions that instructor-led group participants attended) using the Spearman’s rho correlation index.

#### Effectiveness of Self-Taught and Instructor Led MT and the Possible Contribution of Implementation Variables

To assess the effectiveness of self-taught and instructor-led programs in improving participating secondary teachers’ well-being and mental health, we used hierarchical linear regression mixed models on an intention-to-treat basis. These models included teachers and schools (clusters) as random effects, fitting wave and the group-by-wave interaction, and modeling correlations at the school-level but focused on the variance at the teacher-level. The number of schools that entered into the study was 43, with an average of five participating teachers in each school (from a minimum of three to a maximum of nine). We used robust maximum likelihood (sandwich) variance estimates that adjust for within-cluster correlations obtaining cluster robust standard errors (Williams, 2000). The number of clusters needed for this kind of model should be more than 20 (Snijders & Bosker, 2011), the alternative population averaged methods (e.g., those that account for clustering without explicitly splitting the model into multiple levels) are only more advantageous with an average for the cluster sizes less than five (McNeish, 2014), and the hierarchical method we used here functions better in case of cluster size imbalance (Verbeke et al., 2014).

First, we carried out within-group tests contrasting differential scores without including covariates in the models to ascertain possible improvements in the psychological well-being and mental health outcomes in each MT delivery format. We calculated unstandardized regression coefficients (*B*) from complete cases (those who had data at both T0 and T1) and marginal means. Within-group effect sizes were also calculated by correcting for the dependence of the repeated measures (Morris & DeShon, 2002). Standardized effect sizes (*d*) = .20 are usually regarded as small, around .50 as medium, and .80 as large. We then carried out between-group comparisons without including covariates in the models to ascertain differences in the psychological well-being and mental health outcomes by treatment group. Effect sizes for each pairwise comparison between groups were calculated—we used the pooled pretest *SD* to weigh the differences in the prepost scores and to correct for the population estimate (Morris, 2008). After running these tests, we calculated the amount of variation in the prepost intervention dataset that was explained by the variation between clusters by means of the intraclass correlation coefficient (ICC).

Second, we developed sensitivity analyses that were established a priori (Crane et al., 2020) as a way of controlling for the possible effect on results of routine demographics and baseline differences. For that, we estimated models adjusted for the baseline level of the outcome, with participating teacher’s gender, age, and years of teaching experience as covariates. Moreover, we calculated models using imputed data without including covariates as a way of taking into account the effect of attrition. A missing-values analysis was developed using bivariate logistic regressions, including baseline measures as independent variables and missingness as a dependent variable. Multiple imputations of 20 data sets based on chained equations of linear regressions were developed to address missing data using the full sample. The imputation model included all the variables used in the raw analyses and the baseline covariates of adjusted models, cluster size, and those variables related to nonresponse (those variables finally included in the imputation model are specified at the foot of the Supplementary Materials S10 and S13). In addition, models only adjusted for implementation variables such as expectancy, frequency of mindfulness practice, and reading the book, as covariates were carried out (these last models were decided a posteriori).

Finally, we explored how the implementation variables of expectancy, reading the book and weekly days of mindfulness practice might independently impact prepost change in teacher outcomes as a measure of how they might be linked to effectiveness in each delivery format (we did not include credibility in these models as this is a posttest implementation variable). This was carried out using the hierarchical linear regression mixed analytic approach described at the beginning of this section but specifically looking at the potential predictor-by-wave interactions in a within-group comparison (i.e., by comparing pre- and postscores among individuals who completed the same program).

#### Psychological Mechanisms of Change: The Potential Mediating Role of Mindfulness and Self-Compassion Skills

We analyzed (a) the indirect effects of the frequency of mindfulness meditation practice (independent variable) on prepost improvements in the teacher outcomes (dependent variables), through prepost gains in (i) mindfulness or (ii) self-compassion (process measures), for each group of treatment separately. In addition, we explored (b) the indirect relationships between the treatment condition (independent variable) and prepost improvements in the teacher outcomes (dependent variables), through prepost gains in (i) mindfulness or (ii) self-compassion (process measures), using complete cases analysis.

We used a simple mediation path-analytic framework considering the group-level where the random assignment took place in order to account for the clustering of observations. However, because we were interested in the results at the teacher level, we had no theoretical interest in effects on the different levels/cross-level interactions. Thus, in order to prevent possible difficulties related to the absence of a sufficient number of clusters to analyze all the parameters involved in the analysis of random slopes in the mediating models, we used the weighted log-likelihood function. For this we used a sandwich estimator to compute cluster-robust standard errors with the maximum likelihood robust algorithm. This method does not model random effects, but instead makes a small number of assumptions—for example, it does not require the assumption of normality and yields robust estimates of asymptotic covariances of parameter estimates (Preacher et al., 2010). Results of this algorithm provide unstandardized path estimates that can be interpreted identically to single-level methods, but with the benefit that results are adjusted to reflect clustering of observations (McNeish et al., 2017).

**Figure 1 fig1:**
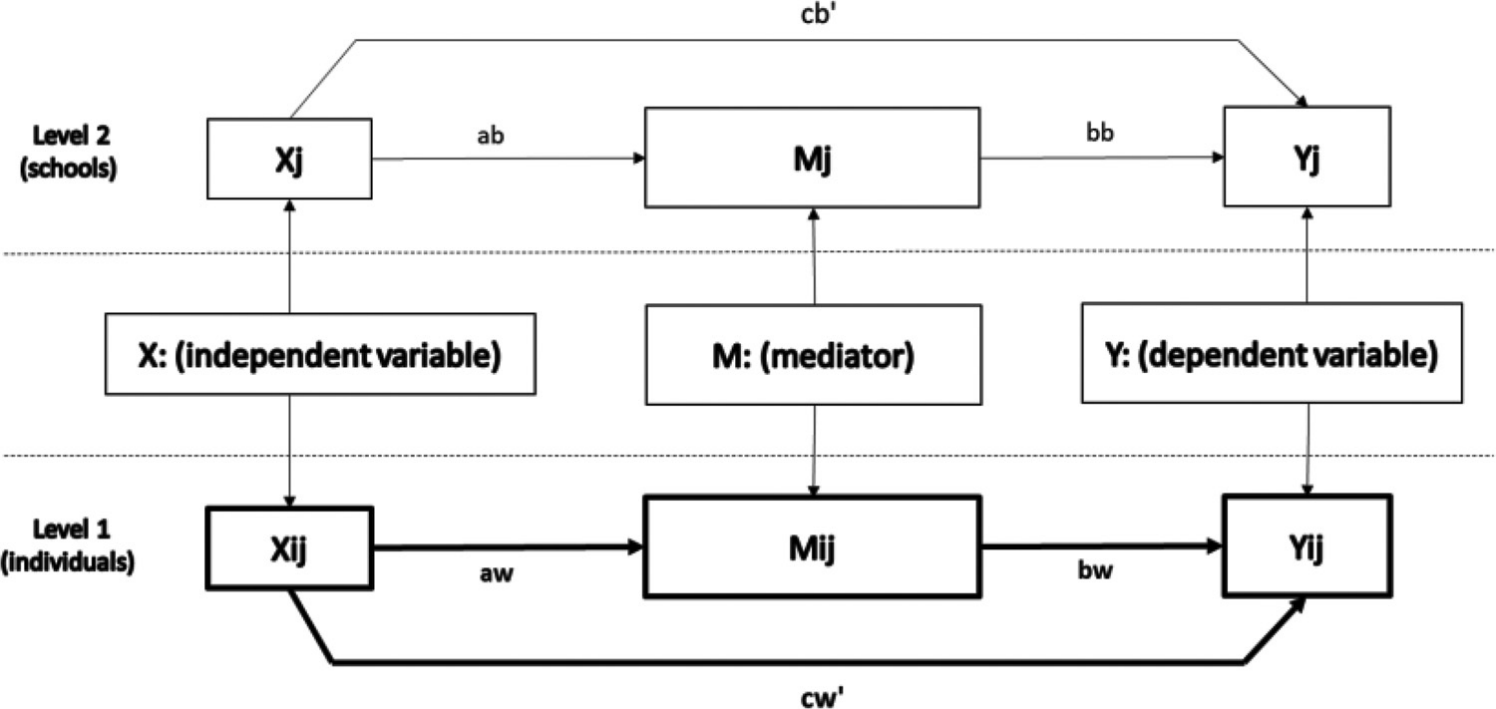
Path-Analytic Mediating Framework Accounting for the Clustering of Observations *Note*. Path-analytic framework considering the group-level where the random assignment took place in order to account for the clustering of observations. Results at the teacher level are adjusted for the clustering of observations and are highlighted because they are the object of interest in the present study. The independent variable (X) is (a) the frequency of mindfulness practice or (b) the treatment condition. The mediator (M) is (a) the FFMQ prepost difference or (b) the SCS prepost difference (simple mediation). The dependent variable is the prepost difference in the corresponding teacher well-being outcome (Y). “aw _*_ bw” = indirect effect through the corresponding mediator. cw' = direct effect after adjusting for the mediating effects.

We calculated *p*-values for each path coefficient (*aw*, *bw*, and *cw’* in Figure 1) using the delta method, but we used the 95% CI for the indirect effect based on a Monte Carlo simulation. This procedure has a better performance to cope with the absence of normality of standard errors of indirect effects (MacKinnon et al., 2004). For that, we estimated the joint distribution of the “*aw*” and “*bw*” slopes using 20,000 random draws from the parameter estimates and their associated asymptotic variances and covariance. Indirect effects are significant when their 95% CI does not include zero. The effect sizes of the mediating models were calculated using *R^2^* as the proportion of the prepost change in the dependent variable that is not associated with the independent variable but is associated with the prepost change in the mediator. This was weighted by the proportion of variance explained in the prepost change in the mediator by the independent variable (MacKinnon, 2008), with values of .00 = null, .14 = small, .39 = medium, and .59 = large effects (Fairchild et al., 2009).[Fig fig1]

The overall *alpha* significance level was set at .05 using a two-sided test. Because the study was exploratory, we did not use corrections for multiple measurements but instead took care to interpret effect sizes and confidence intervals (Feise, 2002). Analyses were performed using the STATA v12.0, Mplus v8.4, R v4.2, and IBM SPSS v26.0 statistical packages.

## Results

### School and Participant Characteristics and Study Flow

**Figure 2 fig2:**
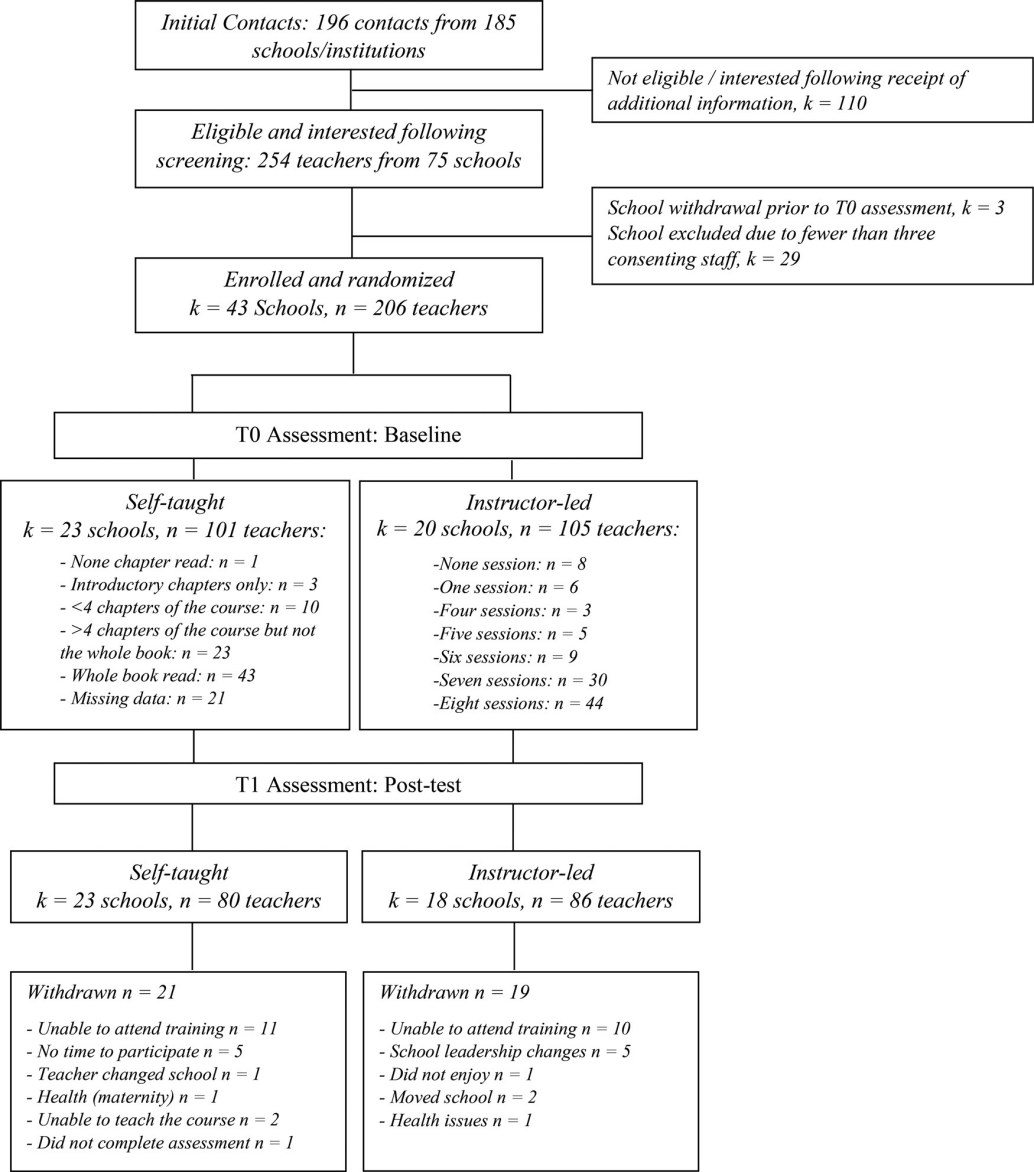
Study Flow of Participants

A total of 43 schools and 206 participating teachers took part in the trial and were randomly allocated to the self-taught (101 participating teachers in 23 schools) or to the instructor-led (105 participating teachers in 20 schools) arm. As can be seen in Figure 2, 41 (95.4*%*) schools and 166 (80.6*%*) participating teachers provided data immediately postintervention (T1) and therefore were included in the complete cases analyses, with a total of 80 (79.2*%*) participating teachers in the self-taught group and 86 (81.9%) in the instructor-led group (χ^2^ = .10; *p* = .752). On the other hand, all 43 schools and 206 participating teachers that started the trial were included in the sensitivity analysis using multiple imputations based on chained equations. Imputations were carried out with a 19.4*%* of missing data (reasons of missingness are provided in Figure 2).[Fig fig2]

Table 1 shows the school and participating teacher baseline characteristics of those who provided data at post intervention. As can be seen, groups only showed significant differences at baseline in median years of teaching, and thus this variable was controlled in subsequent analyses. Significant but small between-group differences also appeared in anxiety and burnout when considering the total sample that started the trial, as can be seen in the Supplementary Material S2. Only gender was a significant predictor of missing data at post intervention, although number of years teaching, the school level of pupil deprivation (measured as the percentage of free school meals), and expectancy showed a trend (for more details see Supplementary Material S8). No other variable was involved in the missing pattern, and thus it was considered to be at random (MAR; White et al., 2011).

### Expectancy and Credibility of Self-Taught and Instructor-Led MTs

There were no significant differences between groups in expectancy toward the program (self-taught: *M* = 7.5; *SD* = 1.7; instructor-led: *M* = 7.8; *SD* = 1.5; *p* < .339). In the self-taught group, expectancy was a predictor of reading the book (*ρ =* .36; *p* = .003), and frequency of practice (*ρ =* .33; *p* = .008). In the instructor-led group, there were no significant associations between expectancy and reading the book (*ρ =* .01; *p* = .972), frequency of practice (*ρ =* .20; *p* = .102), and group sessions attended (*ρ =* .15; *p* = .201). After intervention, credibility was significantly higher in the instructor-led arm than in the self-taught (self-taught: *M* = 7.6, *SD* = 1.7; instructor-led: *M* = 8.6; *SD* = 1.5; *p* < .001).

### Engagement With Self-Taught and Instructor-Led MTs

The median number of days self-taught participating teachers reported practicing mindfulness meditation during the program was four per week (interquartile range: 3 to 5), with a median of five per week (interquartile range: 4 to 6) for the instructor-led group. There were no significant differences between groups in terms of frequency of mindfulness practice (Mann–Whitney z = −1.04; *p* = .300). In the self-taught group—considering those who reported data—43 participating teachers (53.8*%*) read the whole book and 66 (82.5*%*) read > 4 chapters of the course. In the instructor-led group, 46 participating teachers (53.5*%*) read the whole book and 75 (87.2*%*) read > 4 chapters. There were no significant differences between groups in terms of reading the book (Mann–Whitney z = −.33; *p* = .743). Considering all participants who started the MT with instructor, 44 (41.9*%*) completed the whole MT course and 91 (86.7*%*) completed at least half of the course. The median number of group sessions attended by instructor-led participating teachers was seven (interquartile range: 6 to 8).

### Effectiveness of the Self-Taught and Instructor-Led MTs

The raw descriptive data of all the teacher outcomes and mechanisms by arm can be found in the Supplementary Material S9.

#### Within-Group Analyses

The within-group analyses of the self-taught arm based on complete cases (i.e., 23 schools with 80 complete cases) revealed small, but significant prepost improvements in well-being and self-compassion, with moderately small effect sizes (see Table 2). There were no significant effects on burnout, depression, anxiety, stress, or mindfulness. On the other hand, the within-group analyses of the instructor-led arm based on complete cases (i.e., 18 schools with 86 complete cases) showed significant prepost improvements in all the teacher outcomes, with moderate effect sizes (see Table 2).[Table tbl2]

The within-group results obtained from models adjusting for teacher’s gender, age, years of teaching experience and the baseline levels (Supplementary Material S10) were consistent with those obtained from the main analyses, although effect sizes were increased. The within-group results from models adjusting for the implementation variables of expectancy, frequency of mindfulness meditation practice, and reading the book showed similar results to those from the main analyses, and from models adjusting for teacher’s gender, age, years of teaching experience and the baseline levels, but effect sizes decreased notably (Supplementary Material S11). The within-group analyses from imputed models in the self-taught group (i.e., 23 schools with 101 teachers) presented significant improvements in mindfulness, self-compassion and well-being, while in the instructor-led group (i.e., 20 schools with 105 teachers) they showed significant improvements in mindfulness, self-compassion, well-being and perceived stress (Supplementary Material S12).

#### Between-Group Analyses

The between-group complete case analyses (i.e., 41 schools with 166 complete cases) showed there were significant group-by-wave interactions in all outcomes, except for burnout (which showed a trend), with low to moderate educator surveys (ESs), favoring the instructor-led group (see Table 3). The between-school intraclass correlation coefficients ranged between .00 for mindfulness, well-being, stress and anxiety, and .08 (95% CI [.00, .18]) for depression, with values of .06 (95% CI [.00, .20]) for self-compassion, and of .04 (95% CI [.00, .15]) for burnout (see Table 3). Therefore, there was a considerable school clustering effect that was corrected by means of the analytical procedures described above.[Table tbl3]

Adjusted models with complete cases (i.e., 41 schools with 166 complete cases) controlling for teacher’s gender, age, years of teaching experience, and the baseline levels demonstrated quite similar regression coefficients, and significant effects were maintained with effect sizes ranging (Cohen’s *d* absolute value) from .39 to .70 (see Supplementary Material S13). Only the baseline levels were significant covariates in all the adjusted models (*p* < .001), with years of teaching being a significant covariate in the perceived stress model (*B* = −.06; *p* = .032). Adjusted models controlling for expectancy, frequency of mindfulness practice, and reading the book showed significant between-group differences in all the outcomes except burnout, favoring the instructor-led group (Supplementary Material S14). However, effect sizes decreased notably compared with those obtained in the primary analyses (Cohen’s *d* absolute value ranged from .17 to .43). Frequency of mindfulness meditation practice was a significant covariate for all outcomes but burnout, while expectancy was a significant covariate only for mindfulness and self-compassion. Reading the book was not a significant covariate in any of the models analyzed. Imputed models based on chained equations (i.e., 43 schools with 206 teachers) attenuated between-group differences, and only those in self-compassion and perceived stress remained significant, with effect sizes (Cohen’s *d* absolute value) from .14 to .52 (Supplementary Material S15).

#### Analyses of Implementation Variables

The analysis exploring how the implementation variables of expectancy, frequency of mindfulness meditation practice, and reading the book might be linked to effectiveness in each arm (Supplementary Material S16) showed that expectancy had a significant impact on improvements in mindfulness, self-compassion, and well-being in the self-taught arm (i.e., 23 schools with 80 complete cases), while it had a significant impact on mindfulness, well-being, perceived stress, and depression in the instructor-led arm (i.e., 18 schools with 86 complete cases). The implementation variable of frequency of mindfulness meditation practice had significant relationships with outcome improvements in both arms, with the exception of burnout symptoms. Reading the book was not significantly related to teacher outcome improvements in the instructor-led arm, but in the self-taught arm, it was significantly associated (or trended) with improvements in all the outcomes.

### Mediating Role of Mindfulness and Self-Compassion Skills

Frequency of mindfulness meditation practice produced significant indirect effects on all the psychological outcomes in both delivery formats, through the mediating effects of mindfulness (Supplementary Material S17), with small to medium ESs. However, frequency of mindfulness meditation practice had no significant indirect effects on teacher outcomes through self-compassion in either the self-taught arm nor in the instructor-led arm (Supplementary Material S18). Frequency of mindfulness meditation practice was not significantly related to improvements in self-compassion skills in the mediational model, although improvements in self-compassion skills were significantly related to improvements in all the teacher outcomes across both formats of delivering the MT. On the other hand, as can be seen in Table 4, being randomized to the instructor-led arm versus the self-taught arm produced significant indirect effects on all the outcomes through both mindfulness and self-compassion, with small to medium effect sizes.[Table tbl4]

## Discussion

This study explored the relative acceptability, engagement, and effectiveness among secondary school teachers of instructor-led and self-taught MT using the M-FP program (Williams & Penman, 2011). We further explored the possible mechanisms of change involved. Our results suggested that the instructor-led format produced greater improvements in mindfulness, self-compassion, well-being, stress, anxiety, and depression than the self-taught format. There are several possible explanations for the differential effects observed. First, the instructor-led format is additive in that it includes the book used in the self-taught format as well as the eight 90-min instructor-led group sessions. However, we have seen that reading the book only contributed significantly in the self-taught group—in fact, it was the only way of receiving the intervention for that group—while it seemed that for people doing the face-to-face course the book may have been redundant as they received the content in the group sessions. Second, the instructor-led format includes expert support which may provide ways for participants to identify and work with obstacles and difficulties in following the program, modeling skills and consolidating their learning. Third, it has been suggested that group work is important in the MT experience (Irving et al., 2014) and, when learning is social, it significantly enhances the acquisition of new skills (Botella et al., 2009; Cavanagh et al., 2014). According to the self-determination theory, the satisfaction of the need for relatedness could have also led to psychological improvements (Ryan & Deci, 2017; Vansteenkiste et al., 2020). Nevertheless, it is necessary to recognize it is not possible to attribute the superior effects of the instructor-led format to the group experience only. An additional study arm, in which teachers were provided with one-to-one instruction, could provide information on this. However, mindfulness-based programs are usually developed as group programs due to cost-effectiveness concerns (Segal et al., 2013).

### Expectancy, Credibility, and Engagement With the MTs

Both curricula were associated with high and similar ratings of expectancy at the beginning of the program, perhaps reflecting the fact that teachers were self-selected and motivated. In general, expectancy at the beginning of an intervention is associated with compliance (Joyce & Piper, 1998), and this might explain the absence of significant between-group differences in engagement with mindfulness practice and reading the book. The self-taught arm presented a median of 4 days per week of practice and the instructor-led arm showed a median of 5 days per week. Around half of participants from both arms read the whole book, and more than two thirds covered at least half of the sessions in the instructor-led arm. These engagement rates were in the expected range (Emerson et al., 2017; Klingbeil & Renshaw, 2018; Parsons et al., 2017), and were similar to those obtained in previous studies using the same curriculum (Beshai et al., 2016; Lever-Taylor et al., 2014), suggesting that both MTs could be acceptable. Nevertheless, as we have observed, MT suffers from substantial rates of attrition when applied to natural settings (Khoury et al., 2013; Nam & Toneatto, 2016), and failure to consolidate meditation practice undermines the effectiveness of the MT (Crane et al., 2014; Sekhon et al., 2017). Thus, engagement with the MT could be considered appropriate, but there is room for improvement (Kuyken et al., 2008).

A recent study highlighted the need for committed individuals to champion the approach within their schools, with the explicit support of members of the school leadership team; as well as the importance of the initial perceptions of what MT is and why it is being introduced in the school context. Both of these were highlighted as facilitating factors that might support the implementation of MT in educational settings (Wilde et al., 2019).

In other contexts, implementing a virtual community of support through a WhatsApp group or emails offering daily reminders to assist and complete the mindfulness meditation practice with motivating messages, has also been recommended (Montero-Marin et al., 2020). All in all, frequency of days of mindfulness meditation practice had a significant relationship with the majority of teacher outcomes in both delivery formats, which reinforces the idea of frequency of practice as an important implementation variable that might underlie MT effectiveness (Crane et al., 2014; Hawley et al., 2014; Parsons et al., 2017; Segal et al., 2013).

We have seen that credibility values after finishing treatment were high in both intervention groups. Interestingly, although there were no significant between-groups differences in the frequency of mindfulness meditation practice during the program, the self-taught arm showed significantly lower credibility scores than the instructor-led arm. The reasons for this could be related to the beneficial effects produced by the instructor and the group modeling the acquisition of new skills, but this needs to be specifically investigated in future research. In general, credibility after treatment has been observed as being associated with outcomes (Mooney et al., 2014), which aligns with our results as the self-taught group had smaller effects than the instructor-led group.

When training teachers to deliver a MT to secondary pupils, it has been said that the differences in levels of secondary teacher competency achieved between the most intensive training, compared with the more scalable alternatives, might be modest. Economic evaluation suggests that greater intensity could be both more expensive but also more effective than lower intensity, although significant differences have not been observed (Crane et al., 2020). In this sense, the reality that self-taught programs for education may be more sustainable due to challenges of instructor-led programs—which need extensive and intensive mindfulness instructor support in the everyday course of school and teaching life—is a bit of concern, given that the instructor-led program seemed to be superior. Nevertheless, benefits associated with self-help approaches such as access and availability make this format an ongoing area of interest for increasing the mental well-being of teachers, students, and the general population (Cavanagh et al., 2014). Our results suggest that the self-taught program might be indicated for those who cannot access or commit to an instructor-led program, something that is particularly relevant to school teachers who have very full lives during the school academic year. However, more research is needed to examine whether providing minimal but regular (e.g., phone, WhatsApp. or email) contact with those using a self-taught format could make a difference. This has been shown to bring about marked improvements in outcomes for very little added effort (Talbot, 2012). In summary, it seems like these nonspecific implementation factors could have important implications for the MT delivery, and may point toward differences in effectiveness and mechanisms (Stinson et al., 2018).

### Effectiveness of the Self-Taught and Instructor-Led MTs

Our results for the MT instructor-led format are in line with other studies showing medium effects on teacher stress and emotion regulation (Emerson et al., 2017). Other MTs for the general population have also shown similar medium effects in improving quality of life and reducing stress, depression, and anxiety (Khoury et al., 2015). Interestingly, one exception to this pattern of findings was a study of a self-taught mindfulness curriculum for undergraduate and postgraduate students, which demonstrated large effect sizes on mindfulness, self-compassion, satisfaction with life, perceived stress, anxiety, and depressive symptomatology (Lever-Taylor et al., 2014). Nevertheless, participants in this study reported considerable high baseline levels of mental distress and therefore it is possible these considerably larger effects are explainable in terms of participants having greater scope, and potentially motivation, to engage in psychological change processes.

Our study focused on a sample of secondary school teachers whose mental health was in the normal range, according to descriptive data. By focusing on changes in universal mechanisms that underpin mental health, MT might potentially move the whole secondary teacher population toward greater levels of well-being (Feldman & Kuyken, 2019). This is a different approach from one in which psychological therapies target mechanisms maintaining psychopathology (Redelinghuys et al., 2019), and it is consistent with our finding that MT is less effective in improving burnout, which may require a more systemic approach. Nevertheless, observing small effects in burnout as a result of MT is not rare (Hwang et al., 2017; Lomas et al., 2017). It seems like teacher burnout might be improved by using MT but may be in need of interventions not only at the individual level but also organizationally (Klingbeil & Renshaw, 2018; Montero-Marin et al., 2013). In our study, the unusually healthy profile of the teacher sample in terms of burnout (see Supplementary Material S5), might have produced floor effects, although we observed significant within-group improvements on burnout in the instructor-led group, as well as significant indirect effects of frequency of mindfulness meditation practice in both self-taught and instructor led arms.

The instructor-led MT did improve all the teacher outcomes, but, without having an appropriate control group comparator, we do not know to what extent teachers’ mental health would improve spontaneously across the academic year. Nevertheless, it has been observed that teacher stress might be increased by nearly 20% throughout the school academic year (von der Embse & Mankin, 2021). So, considering that this study was carried out in the second half of the school academic year, spontaneous improvements would not be expected. In general, teachers usually report high levels of occupational stress and burnout (Farber, 1991), and have been found to be at high risk of mental health disorders such as anxiety and depression (Stansfeld et al., 2011), with lower levels of well-being when compared with the general working population (Kidger, Stone, et al., 2016). A previous comparable study with more than 500 English secondary teachers (Kidger, Brockman, et al., 2016) showed that the levels of mental health observed were worse (WEMWBS = 47.2; PHQ-9 = 5.8) than those obtained in the present study, considering both MT groups at pre- and postintervention. It has been observed that standard group MTs for this workforce seems to offer similar results to those in other nonclinical adult populations and working professionals, with medium effects (Klingbeil & Renshaw, 2018). However, the self-taught group reached an improvement of less than 3 points in the WEMWBS, and thus the meaning of changes obtained in this group would need to be considered with certain caution (Putz et al., 2012). Thus, more research comparing the MT effectiveness for secondary school teachers, implemented at different school year time points, is necessary to evaluate all these possible effects.

### Potential Mechanisms of Change of the MTs

A prosocial classroom and students’ social and emotional learning have to start with the teachers’ well-being (Jennings & Greenberg, 2009). It has been proposed that both mindfulness and self-compassion skills may be important mechanisms to improve mental health in a range of populations (Gu et al., 2015), and this has specifically been observed in secondary teachers (Roeser et al., 2013). There is a general assumption that the cultivation of mindfulness leads to nonreactive acceptance of one’s experience, disengaging from nonadaptive thoughts, and improving emotion regulation, which in turn lead to positive outcomes (Emerson et al., 2017; Feldman & Kuyken, 2019; Segal et al., 2013; Van der Velden et al., 2015). Our results suggest that the instructor-led arm, compared with the self-taught, was able to indirectly improve teacher outcomes by activating the mediating role of both mindfulness and self-compassion skills. This was also observed by Roeser et al. (2013), when they compared an instructor-led group of MT versus wait-list controls. In addition, we have observed that the implementation variable of frequency of mindfulness meditation practice showed significant indirect effects on teacher outcomes through the mediating role of mindfulness, but not self-compassion, across both the instructor-led and self-taught ways of delivery. Interestingly, improvements in self-compassion were significantly associated with improvements in the teacher outcomes in both self-taught and instructor led groups. We do not know whether other implementation variables that were not measured in the present study could differentially activate self-compassion as a possible mechanisms of change.

The M-FP program book (Williams & Penman, 2011) was originally developed as a self-help approach but has been adapted for use in an instructor-led format. As we have observed, the instructor-led MT seems to enhance the effectiveness of the program in secondary school teachers. It appears to work through mindfulness and self-compassion skills as possible mechanisms of change, when compared with the self-taught format. Nevertheless, it is necessary to study whether differences in effectiveness and mechanisms that vary according to the way MT is delivered, might be explained by other implementation variables such as practice length and depth, program integrity, social aspects of learning, and teacher guiding (Botella et al., 2009; Cavanagh et al., 2014; Segal et al., 2013).

### Study Limitations

The primary aim of this study was to examine the cost-effectiveness and scalability of different models of teacher training (see Crane et al., 2020). Thus, it might be underpowered to fully investigate all of the questions, and the possibility of Type I and II errors might arise. Although the mediation models used were adequate for the exploratory aims proposed here (Montoya & Hayes, 2017), and our findings are broadly in line with previous work (Roeser et al., 2013), future research should use a greater number of time points in adequately powered studies. Also, observed scale scores were modeled, not latent factors purged of unreliability, so larger samples should also be recruited in order to conduct latent modeling where “true” variance and unique effects can be better estimated. The fact that the instructor-led group showed significantly worse baseline scores than the self-taught in both anxiety and burnout, when considering the total sample that started the trial, could facilitate greater improvements in the first group because its possible improvement range was larger. In the absence of a no-treatment control group, differences in effectiveness between the instructor-led and self-taught MT could not be attributed entirely to the MT delivery. This makes it difficult to determine the effect of time alone on changes in teacher outcomes. It might be possible that teacher outcomes tend to improve over the school year so the design used here makes it difficult to evaluate the effectiveness of each active arm. Nevertheless, previous studies suggest teacher outcomes tend to get worse over the course of an academic year (von der Embse & Mankin, 2021). In addition, when considering instructor led and self-taught formats of the M-FP program, the use of wait-list controls has shown promising results with similar outcomes in secondary teachers and undergraduate students (Beshai et al., 2016; Lever-Taylor et al., 2014). Our measure of engagement with mindfulness meditation practice, based only on self-reported frequency of practice (from surveys and diaries), was relatively simple, and therefore, could have been subject to different forms of bias. In addition, one might imagine different home environments that could lead to a different application of mindfulness practice, and, this could vary depending on life stage or family life. Therefore, findings of frequency of practice should be tempered in this regard. A more nuanced measurement, including other aspects such as length and depth of practice, as well as the different conditions in which practice is being carried out, would have been advantageous. Additionally, given our focus on teachers and their workplace well-being, it would have been useful to include more measures of job functioning, as well as school-based mindfulness practice. Finally, the sample comprised of teachers with an interest in social-emotional education generally and MT specifically, as this was part of their preparation to teach MT to their pupils. In this sense, participants may not be representative of the larger population of school teachers, which may explain their generally good mental health and relatively engaged profile. Thus, it is possible that those teachers with the lowest levels of well-being—the ones who in fact would most need the program—were underrepresented. The study is therefore exploratory and hypothesis generating and serves as a base for subsequent future research.

### Implications and Future Directions

This study suggests that both self-taught and instructor-led modes of MT generated similar levels of expectancy at the beginning of the intervention and engagement with the program during its implementation, but the instructor-led format was rated as more credible once the program was finished. This is easy to understand given that the self-taught group showed significant improvements in around 30*%* of the teacher outcomes, while the instructor-led obtained significant improvements in 85*%* of the outcomes. Thus, the more intensive instructor-led MT produced better results but, considering how teaching life is during the course, the self-taught program is likely to be more accessible (although requiring greater self-motivation). This opens new avenues for future studies regarding MT intensity and its cost-effectiveness and sustainability. Previous research has questioned the move toward abbreviating secondary teacher trainings to increase scalability, and on the contrary, suggests that many teachers would require additional support to ensure their competency to deliver MT in the classroom (Crane et al., 2020). Future studies should examine how the particular features of self-taught and inductor-led formats can facilitate adherence to the MT program through other implementation variables that might influence effectiveness and mechanisms. To strengthen this line of research, prospective works should consider longer follow-ups that examine the sustainability of any effects. In this sense, the next phase of research has the promise of outlining which forms of MT delivery improve which psychological and behavioural outcomes, how, for whom, and through what mechanisms of action. Such knowledge would be the basis for enhancing accessibility and effectiveness of interventions to improve the mental health and functioning of secondary teachers.

## Supplementary Material

10.1037/edu0000542.supp

## Figures and Tables

**Table 1 tbl1:** Baseline School and Participant Characteristics of Complete Cases

School/participant variables	Total group	Self-taught	Instructor-led	*p*
School characteristics	*k* = 41	*k* = 23	*k* = 18	
Percentage free school meals, median (IQR)	19.3 (12.9, 39.1)	15.4 (11.4, 35.2)	24.1 (16.4, 45.8)	.169
State schools, *n* (%)	36 (88)	21 (91)	15 (83)	.638
Large schools, *n* (%)	22 (54)	13 (57)	9 (50)	.920
OFSTED good/outstanding (state schools), *n* (%)	27 (75)	16 (76)	11 (73)	.807
More than five teachers recruited, *n* (%)	21 (51)	11 (48)	10 (55)	.627
Participant characteristics	*n* = 166	*n* = 80	*n* = 86	
Age, *M* (*SD*)	38.8 (9.1)	39.6 (8.6)	38.0 (9.6)	.261
Female, *n* (%)	134 (81)	64 (80)	70 (81)	.999
Marital status (married or with partner), *n* (%)	120 (72)	58 (73)	62 (72)	.920
Number of years teaching, *Mdn* (IQR)	11 (6, 18)	12 (7, 19)	8 (4, 17)	.009
FFMQ-SF, *M* (*SD*)	51.5 (6.8)	51.4 (6.6)	51.6 (7.0)	.883
SCS-SF, *M* (*SD*)	3.2 (0.8)	3.2 (0.9)	3.2 (0.8)	.999
WEMWBS, *M* (*SD*)	49.1 (7.2)	49.1 (7.4)	49.0 (7.1)	.972
PSS, *M* (*SD*)	15.7 (7.2)	15.0 (7.1)	16.3 (7.3)	.232
PHQ-9, *M* (*SD*)	4.8 (3.7)	4.6 (3.8)	4.9 (3.5)	.469
GAD-7, *M* (*SD*)	4.3 (4.4)	3.8 (4.4)	4.7 (4.3)	.181
MBI-ÉS, *M* (*SD*)	38.7 (17.7)	36.6 (18.5)	40.3 (16.3)	.173
*Note*. Complete cases are those providing data on outcomes at T1 and thus included in the analyses. Three schools in each of the instructor-led and self-help groups have missing data on the percentage of pupils claiming free school meals. Data are complete, in both groups, for all other baseline variables included. IQR = interquartile range; OFSTED = Office for Standards in Education, Children’s Services, and Skills; FFMQ-SF = Five Facets Mindfulness Questionnaire Short Form; SCS-SF = Self-Compassion Scale Short Form; WEMWBS: Warwick-Edinburgh Mental Well-Being Scale; PSS = Perceived Stress Scale; PHQ-9 = Patient Health Questionnaire-9; GAD-7 = General Anxiety Disorder-7; MBI-ES = Maslach Burnout Inventory-Educators Survey.				

**Table 2 tbl2:** Self-Taught and Instructor-Led Within-Group Complete Cases Analysis

Group/variable	Time	*M* (*SD*)	*d*	*B* [95% CI]	*p*
Self-taught (*n* = 80)					
FFMQ-SF	T0	51.51 (6.71)			
	T1	52.43 (8.68)	.18	0.92 [−0.49, 2.33]	.202
SCS-SF	T0	3.10 (0.97)			
	T1	3.28 (0.90)	.25	0.18 [0.02, 0.34]	.024
WEMWBS	T0	49.41 (7.01)			
	T1	51.00 (6.85)	.25	1.60 [0.01, 3.19]	.048
PSS	T0	14.78 (6.93)			
	T1	14.84 (7.77)	.01	0.06 [−1.62, 1.73]	.944
PHQ-9	T0	4.43 (4.14)			
	T1	4.53 (6.11)	.02	0.10 [−1.32, 1.53]	.890
GAD-7	T0	3.62 (3.78)			
	T1	3.93 (5.06)	.08	0.32 [−0.68, 1.32]	.532
MBI-ES	T0	34.42 (19.50)			
	T1	34.23 (18.69)	−.01	−0.19 [−3.24, 2.85]	.902
Instructor-led (*n* = 86)					
FFMQ-SF	T0	51.10 (5.37)			
	T1	54.98 (4.55)	.70	3.88 [2.57, 5.20]	<.001
SCS-SF	T0	3.00 (0.69)			
	T1	3.59 (0.59)	.79	0.59 [0.42, 0.76]	<.001
WEMWBS	T0	48.77 (5.21)			
	T1	53.31 (5.18)	.90	4.53 [3.37, 5.70]	<.001
PSS	T0	16.33 (5.29)			
	T1	12.75 (4.32)	−.67	−3.58 [−4.90, −2.26]	<.001
PHQ-9	T0	4.95 (3.74)			
	T1	3.30 (3.01)	−.43	−1.65 [−2.37, −0.93]	<.001
GAD-7	T0	4.87 (3.00)			
	T1	3.32 (2.43)	−.49	−1.54 [−2.22, −0.87]	<.001
MBI-ES	T0	41.07 (13.91)			
	T1	36.95 (19.48)	−.35	−4.11 [−7.15, −1.08]	.008
*Note*. FFMQ-SF = Five Facets Mindfulness Questionnaire Short Form; SCS-SF = Self-Compassion Scale Short Form; WEMWBS: Warwick-Edinburgh Mental Well-Being Scale; PSS = Perceived Stress Scale; PHQ-9 = Patient Health Questionnaire-9; GAD-7 = General Anxiety Disorder-7; MBI-ES = Maslach Burnout Inventory-Educators Survey. Descriptive are marginal *means* and *SD*s. *d* = Cohen’s d effect size using marginal *means* and *SD*s; *B* = unstandardized regression coefficient using mixed models with subjects and schools as random effects. Complete cases analyses (i.e., self-taught: *k* = 23 schools; instructor-led: *k* = 18 schools).					

**Table 3 tbl3:** Between-Group (Group-by-Wave) Comparison of Mechanisms and Outcomes

		Self-taught(*n* = 80)	Instructor-led (*n* = 86)				
Variable	Time	*M* (*SD*)	*M* (*SD*)	ICC	*d*	*B* [95% CI]	*p*
FFMQ-SF	T0	51.51 (6.71)	51.10 (5.37)				
	T1	52.43 (8.68)	54.98 (4.55)	.00	.49	2.97 [1.04, 4.90]	.003
SCS-SF	T0	3.10 (0.97)	3.00 (0.69)				
	T1	3.28 (0.90)	3.59 (0.59)	.06	.49	0.41 [0.18, 0.64]	.001
WEMWBS	T0	49.41 (7.02)	48.77 (5.21)				
	T1	51.00 (6.85)	53.31 (5.18)	.00	.48	2.94 [0.97, 4.92]	.003
PSS	T0	14.78 (6.93)	16.33 (5.29)				
	T1	14.84 (7.77)	12.75 (4.32)	.00	−.59	−3.64 [−5.78, −1.50]	.001
PHQ-9	T0	4.43 (4.14)	4.95 (3.74)				
	T1	4.53 (6.11)	3.30 (3.01)	.08	−.44	−1.75 [−3.34, −0.15]	.032
GAD-7	T0	3.61 (3.78)	4.87 (3.00)				
	T1	3.93 (5.06)	3.32 (2.43)	.00	−.55	−1.86 [−3.07, −0.66]	.003
MBI-ES	T0	34.42 (19.53)	41.07 (13.91)				
	T1	34.23 (18.70)	36.95 (15.46)	.04	−.24	−3.92 [−8.22, 0.37]	.074
*Note*. FFMQ-SF = Five Facets Mindfulness Questionnaire Short Form; SCS-SF = Self-Compassion Scale Short Form (excluding the “mindfulness” facet); WEMWBS = Warwick-Edinburgh Mental Well-Being Scale; PSS = Perceived Stress Scale; PHQ-9 = Patient Health Questionnaire-9; GAD-7 = General Anxiety Disorder-7; MBI-ES = Maslach Burnout Inventory-Educators Survey. Descriptive data are marginal *means* and *SD*s. ICC = intraclass correlation coefficient to measure the amount of variation in the prepost intervention dataset that was explained by the variation between schools (clusters).;*d* = Cohen’s *d* effect size using marginal means and *SD*s; *B* = unstandardized regression coefficient using hierarchical mixed models with subjects and schools (clusters) as random effects. Complete cases analysis (i.e., self-taught: *k* = 23 schools; instructor-led: *k* = 18 schools).							

**Table 4 tbl4:** Path Estimates and Indirect Effects of Group Allocation Through FFMQ (i) and SCS (ii) on Outcomes

Mediator/DV	*R^2^*	*aw* (*SE*)	*p*	*bw* (*SE*)	*p*	*cw’* (*SE*)	*p*	*IE*s *MC* [95% CI]
FFMQ-SF (i)								
WEMWBS	.25	2.72 (0.99)	.006	0.50 (0.08)	<.001	1.32 (0.97)	.173	1.36 [0.39, 2.51]
PSS	.19	2.72 (0.99)	.006	−0.39 (0.09)	<.001	−2.48 (1.11)	.026	−1.06 [−2.08, −0.26]
PHQ-9	.17	2.72 (0.99)	.006	−0.22 (0.05)	<.001	−1.07 (0.78)	.167	−0.60 [−1.15, −0.15]
GAD-7	.13	2.72 (0.99)	.006	−0.20 (0.06)	.001	−1.14 (0.62)	.066	−0.54 [−1.11, −0.13]
MBI-ES	.22	2.72 (0.99)	.006	−1.01 (0.19)	<.001	−0.31 (2.09)	.882	−2.74 [−5.18, −0.72]
SCS-SF (ii)								
WEMWBS	.23	0.39 (0.12)	.001	4.17 (0.74)	<.001	1.05 (1.09)	.336	1.63 [0.72, 2.74]
PSS	.21	0.39 (0.12)	.001	−3.58 (0.90)	<.001	−2.13 (1.15)	.063	−1.40 [−2.52, −0.52]
PHQ-9	.16	0.39 (0.12)	.001	−1.89 (0.49)	<.001	−0.93 (0.82)	.009	−0.74 [−1.33, −0.27]
GAD-7	.10	0.39 (0.12)	.001	−1.45 (0.50)	.004	−1.11 (0.66)	.094	−0.57 [−1.11, −0.15]
MBI-ES	.24	0.39 (0.12)	.001	−9.05 (1.55)	<.001	−0.52 (2.06)	.802	−3.55 [−5.89, −1.59]
*Note*. DV = dependent variable; *aw* = unstandardized estimated of path “*aw*” (Figure 1); *bw* = unstandardized estimated of path “*bw*” (Figure 1); *cw’* = unstandardized direct effects after controlling for the indirect effects (Figure 1); *SE* = standard error; *IE*s = indirect effects (95% confidence interval for the indirect effect based on a Monte Carlo simulation of the joint distribution of the corresponding slopes using 20,000 random draws from the parameter estimates and their associated asymptotic variances and covariance); FFMQ-SF = Five Facet Mindfulness Questionnaire Short Form; SCS-SF = Self-Compassion Scale Sort Form; WEMWBS = Warwick-Edinburgh Mental Well-Being Scale; PSS = Perceived Stress Scale; PHQ-9 = Patient Health Questionnaire-9; GAD-7 = General Anxiety Disorder-7; MBI-ES = Maslach Burnout Inventory-Educators Survey. Complete cases analysis (i.e. 41 schools with 166 complete cases).
