# Supplementary material for: Teachers “Finding Peace in a Frantic World”: An Experimental Study of Self-Taught and Instructor-Led Mindfulness Program Formats on Acceptability, Effectiveness, and Mechanisms
Source: J Educ Psychol. 2021 Oct 18;113(8):1689–708. doi: 10.1037/edu0000542 (PMC8647626; doi:10.1037/edu0000542)
Supplement: Supplementary file 1 [file EDU-2020-0240_Supplemental_Materials.docx]

**Teachers ‘Finding Peace in a Frantic World’: An experimental study of self-taught and instructor-led mindfulness program formats on acceptability, effectiveness and mechanisms**

**Supplementary Materials**

-*Supplementary Material S1*: School/teacher inclusion/exclusion criteria.

-*Supplementary Material S2*: Baseline school/participant characteristics of the total sample.

-*Supplementary Material S3*: Details of the “Finding Peace in a Frantic World” program.

-*Supplementary Material S4*: Good practice guidelines for mindfulness teachers and mindfulness instructor responsibilities to be included in the trial.

-*Supplementary Material S5*: Fit indices for the scales used.

-*Supplementary Material S6*: Correlations between sub-factors of scales.

-*Supplementary Material S7*: Correlations between total scores of all scales.

-*Supplementary Material S8*: Logistic regression models to assess baseline variables predicting missing outcome data.

-*Supplementary material S9*: Raw descriptive data by arm.

-*Supplementary Material S10*: Self-taught and instructor-led within-group sensitivity analysis of mechanisms and outcomes adjusted for teacher’s gender, age, years of teaching experience and the baseline levels.

-*Supplementary Material S11*: Self-taught and instructor-led within-group analysis adjusted for expectancy, reading the book and weekly days of practice for the sensitivity analysis of mechanisms and outcomes.

-*Supplementary Material S12*: Self-taught and instructor-led within-group analysis with imputed values for the sensitivity analysis of mechanisms and outcomes.

-*Supplementary Material S13*: Adjusted models controlling for teacher’s gender, age, years of teaching experience and the baseline levels for the between-group sensitivity analysis of mechanisms and outcomes.

-*Supplementary Material S14*: Adjusted models controlling for expectancy, reading the book and weekly days of practice for the between-group sensitivity analysis of mechanisms and outcomes.

-*Supplementary Material S15*: Imputed between-group sensitivity analysis of mechanisms and outcomes.

-*Supplementary Material S16*: Implementation variables as predictors of outcome improvements in the self-taught and instructor-led ways of delivering the mindfulness program.

-*Supplementary Material S17*: Path estimates and indirect effects of weekly practice through FFMQ (i) on teacher outcomes.

-*Supplementary Material S18*: Path estimates and indirect effects of weekly practice through SCS (ii) on teacher outcomes.

Supplementary Material S1: School/teacher inclusion/exclusion criteria

| School | Inclusion | (a) Willing to release participating staff for training and to subsequently timetable each participating teacher to deliver the 8-week MT program to at least one class of pupils  (b) Provided headteacher informed consent |
| --- | --- | --- |
|  | Exclusion | (a) Judged inadequate at their last government inspection  (b) Had only an interim head-teacher in post  (c) Had delivered an MT program to their pupils as part of their general program in the previous 12 months  (d) Located in a region that was so geographically remote that an appropriately trained mindfulness instructor could not be identified to deliver the personal mindfulness training to participant teachers within the school  (e) Unable to identify three or more participating teachers |
| Teacher | Inclusion | (a) Held qualified teacher status or, if unqualified, had at least 5 years teaching experience  (b) Were willing and able to undertake personal mindfulness training and to deliver the MT program to their pupils  (c) Provided informed consent |
|  | Exclusion | (a) Were planning to leave the teaching profession within the next 12-18 months  (b) Were on a temporary contract  (c) Had completed a personal mindfulness course in the previous 12 months  (d) Had previously trained to deliver MT to others |

Supplementary Material S2: Baseline school/participant characteristics of the total sample

| School/Participant variables | Total | Self-taught | Instructor-led | p |
| --- | --- | --- | --- | --- |
| *School characteristics* | *K* *= 43* | *K* *= 23* | *K = 20* |  |
| Percentage free school meals, median (IQR) | 19.3 (13.1, 37.9) | 15.4 (11.9, 32.2) | 22.9 (16.4, 39.1) | .177 |
| State schools, n (%) | 38 (88) | 21 (91) | 17 (85) | .650 |
| Large schools, n (%) | 23 (54) | 13 (57) | 10 (50) | .920 |
| OFSTED Good/Outstanding (state schools only), n (%) | 28 (74) | 16 (76) | 12 (71) | .740 |
| More than 5 teachers recruited, n (%) | 22 (51) | 11 (48) | 11 (55) | .863 |
| *Participant characteristics* | *N = 206* | *N = 101* | *N = 105* |  |
| Age in years, mean (SD) | 39.0 (9.0) | 40.1 (8.6) | 38.0 (9.3) | .094 |
| Female, n (%) | 160 (77) | 80 (79) | 80 (76) | .729 |
| Marital status (married or with a partner), n (%) | 153 (74) | 73 (72) | 80 (76) | .632 |
| Number of years teaching, median (IQR) | 11 (6, 18) | 13 (8, 19) | 10 (5, 17) | .007 |
| FFMQ-SF, mean (SD) | 51.3 (7.0) | 51.5 (7.3) | 51.1 (6.8) | .668 |
| SCS-SF, mean (SD) | 3.2 (0.8) | 3.2 (0.8) | 3.1 (0.8) | .657 |
| WEMWBS, mean (SD) | 49.1 (7.4) | 49.4 (7.5) | 48.8 (7.3) | .538 |
| PSS, mean (SD) | 15.6 (7.1) | 14.8 (7.0) | 16.3 (7.2) | .118 |
| PHQ-9, mean (SD) | 4.7 (3.7) | 4.5 (3.8) | 4.9 (3.7) | .339 |
| GAD-7, mean (SD) | 4.3 (4.3) | 3.6 (4.2) | 4.9 (4.3) | .035 |
| MBI-ES, mean (SD) | 37.9 (17.6) | 34.5 (17.7) | 41.1 (16.9) | .007 |

Three schools in each of the instructor-led and self-help groups have missing data on the percentage of pupils claiming free school meals. Data is complete, in both groups, for all other baseline variables included. FFMQ-SF: Five Facets Mindfulness Questionnaire Short Form. SCS-SF: Self-Compassion Scale Short Form. WEMWBS: Warwick-Edinburgh Mental Well-Being Scale. PSS: Perceived Stress Scale. PHQ-9: Patient Health Questionnaire-9. GAD-7: General Anxiety Disorder-7. MBI-ES: Maslach Burnout Inventory-Educators Survey.

Supplementary Material S3: Details of the “Finding Peace in a Frantic World” program

| **Timeline and content** | **Themes** | **Practices** |
| --- | --- | --- |
| Week 1: Waking up to the autopilot | -What is mindfulness?  -Modes of mind: thinking vs sensing; doing vs being  -Waking up from autopilot: “the raisin” exercise  -Stabilizing attention | -Mindfulness of body and breath meditation  -Mindful awareness of a routine activity feeling your body  -Eating mindfully  -Habit releaser |
| Week 2: Keeping the body in mind | -Practising embodied presence, body as refuge and radar  -Appreciation, savoring and gratitude  -Anchoring and grounding  -Mindful listening and speaking  -Reintegrating mind and body | -Body scan  -Ten finger gratitude  -Appreciation here and now  -Keeping the body in mind when speaking and listening  -Routine activity  -Habit releaser |
| Week 3: The mouse in the maze | -Mindfulness in daily life  -Bringing mindfulness into movement  -Breathing spaces  -Becoming aware of striving  -Recognizing your limits | -Mindful movement meditation  -Breath and body meditation  -Breathing space  -Walking practice  -Habit releaser |
| Week 4: Moving beyond the rumour mill | -Relating differently to thoughts and worries  -Thoughts are mental events (come and go), not facts  -We are not our thoughts  -What perspective can my ‘wise mind’ offer on these thoughts and this situation? | -Breath and body meditation  -Sounds and thoughts meditation  -Breathing space  -Habit releaser |
| Week 5: Turning towards difficulties –from reacting to responding | -Exploring difficulty by turning towards difficult sensations and befriending through body  -Reviewing unpleasant events  -Compassionate responding rather than reacting | -Breath and body meditation  -Sounds and thoughts meditation  -Exploring difficulty meditation - cultivating the capacity to respond  -Breathing space  -Habit releaser |
| Week 6: Practising kindness | -Kindness and befriending –random acts of kindness  -Recognising and disengaging from unhelpful self-critical habits  -Growing to love, respect, and honour yourself and others | -Befriending meditation -treating yourself with kindness  -Mindfulness of body and breath meditation  -Breathing space  -Habit releaser (e.g. random acts of kindness) |
| Week 7: When did you stop dancing? | -How to spend time wisely and make choices that support wellbeing  -Rebalancing activities to support well-being: nourishing vs depleting activities  -Cultivating a different relationship to the world | -Rebalancing exercise  -Twenty minute meditation of your choice (nourishing)  -Breathing space + action step |
| Week 8: Your wild and precious life | -“Weaving your parachute” –what are you going to practise?  -Weave what has been learned into a new and sustainable long-term set of practices  -Deciding for yourself which practices you need and for how long to practice  -Maintain formal mindfulness practice  -Tips for using mindfulness in your daily life  -Begin each day with mindfulness  -Increase your level of exercise | -Optimize breathing space  -Recognising and befriending feelings  -Bring mindfulness to daily activities  -Breath and ground attention in the lower half of the body |

Supplementary Material S4: Good practice guidelines for mindfulness teachers and mindfulness instructor responsibilities to be included in the trial

| 1. Good Practice Guidelines for Mindfulness Teachers (http://mindfulnessteachersuk.org.uk/#guidelines ) 2. Mindfulness-based teacher training with a supervised pathway over a minimum of 12 months. 3. Professional qualification in mental or physical health care, education or social care 4. Knowledge and experience of the (teaching) population. 5. Knowledge of relevant underlying psychological processes. 6. Commitment to a personal mindfulness daily practice and participation in annual meditation retreats; engagement to develop mindfulness-based teaching practice by contacts with other practitioners and teachers as well as regular supervision with an experienced mindfulness-based teacher. 7. Commitment to ongoing development as a teacher through further training. 8. Adherence to the ethical framework appropriate to the teacher’s professional background and working context. |
| --- |
| 1. Mindfulness instructor responsibilities to be included in the trial 2. to provide dates and times of availability 3. to provide geographical regions within which teaching would be feasible 4. to agree to teach at least one course, if offered, that matched time and location availability 5. to attend a two day standardisation training course 6. to participate in a conference call immediately prior to teaching 7. to participate in bi-weekly 1 hour supervision conference calls during the period of teaching 8. to maintain confidentiality in line with good clinical practice guidelines 9. to keep a participant register and feedback attendance information to the research team on a weekly basis 10. to inform the research team of any difficulties arising during delivery of the classes so that these can be addressed promptly 11. to be an ambassador for the research project and for mindfulness within participating schools (e.g. by being flexible so that classes place a minimal burden on schools, by answering any questions or concerns where possible, or by feeding back questions/concerns to the research team so that these can be addressed). |

Supplementary Material S5: Fit indices obtained for the scales used

| Scale |  | χ^2^/df | CFI | TLI | RMSEA | SRMR |
| --- | --- | --- | --- | --- | --- | --- |
| Expectations ^a^ |  | 1.83 | .99 | .98 | .07 | .02 |
| Credibility ^a^ |  | 1.62 | .99 | .99 | .06 | .01 |
| FFMQ-SF ^b^ |  | 1.47 | .95 | .94 | .05 | .07 |
| SCS-SF ^c^ |  | 1.72 | .98 | .93 | .06 | .03 |
| WEMWBS ^a^ |  | 1.98 | .94 | .92 | .07 | .05 |
| PSS ^a^ |  | 2.43 | .94 | .92 | .08 | .04 |
| PHQ-9 ^a^ |  | 1.48 | .95 | .94 | .05 | .06 |
| GAD-7 ^a^ |  | 2.00 | .98 | .96 | .07 | .03 |
| MBI-ES ^d^ |  | 1.74 | .92 | .90 | .06 | .06 |

Expectations were measured at the second week of the intervention. Credibility was assessed immediately post intervention. FFMQ-SF: Five Facets Mindfulness Questionnaire Short Form (pre-intervention). SCS-SF: Self-Compassion Scale Short Form (pre-intervention). WEMWBS: Warwick-Edinburgh Mental Well-Being Scale (pre-intervention). PSS: Perceived Stress Scale (pre-intervention). PHQ-9: Patient Health Questionnaire-9 (pre-intervention). GAD-7: General Anxiety Disorder-7 (pre-intervention). MBI-ES: Maslach Burnout Inventory-Educators Survey (pre-intervention). CFI= comparative fit index. TLI= Tucker Lewis Index. RMSEA= root means square error of approximation. SRMR= Standardized Root Mean Square Residual. ^a^ One-factor solution using confirmatory factor analysis and the maximum likelihood robust estimator. ^b^ One-second order factor model using confirmatory factor analysis and the maximum likelihood robust estimator. ^c^ Bifactor model using confirmatory factor analysis and the maximum likelihood robust estimator after removing the “mindfulness” items to avoid redundant item content with the FFMQ-SF. ^d^ Bifactor model using confirmatory factor analysis.

Supplementary Material S6: Correlations between sub-factors of scales

**Correlations between the FFMQ total and sub-scales**

|  | TOTAL | Observing | Describing | Awareness | Non-judging | Non-reactivity |
| --- | --- | --- | --- | --- | --- | --- |
| TOTAL | 1 | .54 (<.001) | .54 (<.001) | .77 (<.001) | .77 (<.001) | .81 (<.001) |
| Observing | .86 (<.001) | 1 | .34 (<.001) | .35 (<.001) | .23 (<.001) | .36 (<.001) |
| Describing | .48 (<.001) | .38 (<.001) | 1 | .32 (<.001) | .29 (<.001) | .30 (<.001) |
| Awareness | .75 (<.001) | .59 (<.001) | .33 (<.001) | 1 | .45 (<.001) | .45 (<.001) |
| Non-judging | .74 (<.001) | .52 (<.001) | .35 (<.001) | .45 (<.001) | 1 | .54 (<.001) |
| Non-reactivity | .94 (<.001) | .75 (<.001) | .36 (<.001) | .63 (<.001) | .65 (<.001) | 1 |

Total and sub-factors were calculated by sum-scores. Values are Pearson’s correlations (p-values in brackets). T0: values above the diagonal (n = 206). T1: values below the diagonal (n = 166).

**Correlations between the SCS total and sub-scales**

|  | TOTAL | Self-kindness | Common Humanity | Self-judgement | Isolation | Over-identification |
| --- | --- | --- | --- | --- | --- | --- |
| TOTAL | 1 | .77 (<.001) | .76 (<.001) | .84 (<.001) | .86 (<.001) | .83 (<.001) |
| Self-kindness | .77 (<.001) | 1 | .57 (<.001) | .60 (<.001) | .53 (<.001) | .54 (<.001) |
| Common Humanity | .71 (<.001) | .51 (<.001) | 1 | .49 (<.001) | .53 (<.001) | .49 (<.001) |
| Self-judgement | .83 (<.001) | .58 (<.001) | .46 (<.001) | 1 | .66 (<.001) | .63 (<.001) |
| Isolation | .82 (<.001) | .49 (<.001) | .46 (<.001) | .58 (<.001) | 1 | .71 (<.001) |
| Over-identification | .90 (<.001) | .61 (<.001) | .52 (<.001) | .73 (<.001) | .74 (<.001) | 1 |

Total and sub-factors were calculated by sum-scores. Values are Pearson’s correlations (p-values in brackets). T0: values above the diagonal (n = 206). T1: values below the diagonal (n = 166). The negatively valenced items‒those included in self-judgement, isolation, and over-identification‒were reversed to calculate the total SCS score.

**Correlations between the MBI-ES total and sub-scales**

|  | M (SD) | TOTAL | Exhaustion | Depersonalisation | (Lack of) Personal Accomplishment |
| --- | --- | --- | --- | --- | --- |
| TOTAL |  | 1 | .93 (<.001) | .79 (<.001) | .65 (<.001) |
| Exhaustion | 23.73 (11.10) | .89 (<.001) | 1 | .65 (<.001) | .38 (<.001) |
| Depersonalisation | 4.10 (4.84) | .74 (<.001) | .55 (<.001) | 1 | .34 (<.001) |
| (Lack of) Personal Accomplishment | 38.40 (6.72) | .62 (<.001) | .26 (<.001) | .30 (<.001) | 1 |

Total and sub-factors were calculated by sum-scores. Values are Pearson’s correlations (p-values in brackets). T0: values above the diagonal (n = 206). T1: values below the diagonal (n = 166). Descriptive values consider the total group at baseline (n = 206). For norm comparisons go to the original manual (Maslach et al. 1996).

Supplementary Material S7: Correlations between total scores of all scales

**Correlations between variables**

|  | Ex/Cr | FFMQ | SCS-SF | WEM | PSS | PHQ-9 | GAD-7 | MBI-ES |
| --- | --- | --- | --- | --- | --- | --- | --- | --- |
| Ex/Cr | 1 | .40 (<.001) | .36 (<.001) | .34 (<.001) | -.27 (<.001) | -.22 (.005) | -.21 (.008) | -.26 (.001) |
| FFMQ-SF | .21 (.010) | 1 | .75 (<.001) | .65 (<.001) | -.62 (<.001) | -.51 (<.001) | -.50 (<.001) | -.55 (<.001) |
| SCS-SF | .14(.098) | .66 (<.001) | 1 | .67 (<.001) | -.64 (<.001) | -.53 (<.001) | -.50 (<.001) | -.53 (<.001) |
| WEMWBS | .10 (.234) | .55 (<.001) | .58 (<.001) | 1 | -.74 (<.001) | -.68 (<.001) | -.65 (<.001) | -.57 (<.001) |
| PSS | -.10 (.256) | -.48 (<.001) | -.54 (<.001) | -.77 (<.001) | 1 | .62 (<.001) | .72 (<.001) | .55 (<.001) |
| PHQ-9 | -.02 (.806) | -.39 (<.001) | -.45 (<.001) | -.63 (<.001) | .68 (<.001) | 1 | .72 (<.001) | .51 (<.001) |
| GAD-7 | .05 (.572) | -.38 (<.001) | -.49 (<.001) | -.59 (<.001) | .69 (<.001) | .80 (<.001) | 1 | .50 (<.001) |
| MBI-ES | -.08 (.344) | -.34 (<.001) | -.47 (<.001) | -.57 (<.001) | .55 (<.001) | .53 (<.001) | .54 (<.001) | 1 |

Factors were calculated by sum-scores. Values are Pearson’s correlations (p-values in brackets). Ex: expectations (second week of the intervention, values below the diagonal). Cr: credibility (post-intervention, values above the diagonal). FFMQ-SF: Five Facets Mindfulness Questionnaire-Short Form. SCS-SF: Self-Compassion Scale-Short Form. WEMWBS: Warwick-Edinburgh Mental Well-Being Scale. PSS: Perceived Stress Scale. PHQ-9: Patient Health Questionnaire-9. GAD-7: General Anxiety Disorder-7. MBI-ES: Maslach Burnout Inventory-Educators Survey. Values are Pearson’s correlations (p-values in brackets). T0: below the diagonal. T1: above the diagonal.

Supplementary Material S8: Logistic regression models to assess baseline variables predicting missing outcome data

| *Characteristics at baseline* | OR | 95% CI | p |
| --- | --- | --- | --- |
| Age | 1.02 | 0.98 to 1.06 | .367 |
| Gender, female | 0.44 | 0.21 to 0.94 | .035 |
| Marital status, married or with partner | 1.81 | 0.75 to 4.37 | .189 |
| Number of years teaching | 1.04 | 1.00 to 1.08 | .074 |
| Percentage free school meals | 0.98 | 0.95 to 1.00 | .060 |
| Type of school, state | 1.31 | 0.56 to 3.07 | .532 |
| School size, large | 0.85 | 0.42 to 1.69 | .637 |
| OFSTED Good/Outstanding | 0.82 | 0.40 to 1.71 | .602 |
| No. of participating teachers in school, more than 5 | 0.92 | 0.45 to 1.88 | .817 |
| Group, self-taught | 1.19 | 0.60 to 2.37 | .625 |
| Expectancy | 0.77 | 0.57 to 1.04 | .081 |
| FFMQ-SF | 0.98 | 0.93 to 1.03 | .365 |
| FFMQ-SF | 0.98 | 0.93 to 1.03 | .365 |
| SCS-SF | 0.85 | 0.68 to 1.05 | .137 |
| WEMWBS | 1.00 | 0.96 to 1.05 | .910 |
| PSS | 0.99 | 0.94 to 1.04 | .622 |
| PHQ-9 | 0.97 | 0.88 to 1.07 | .527 |
| GAD-7 | 0.98 | 0.91 to 1.07 | .707 |
| MBI-ES | 0.99 | 0.96 to 1.01 | .156 |

FFMQ-SF: Five Facets Mindfulness Questionnaire Short Form. SCS-SF: Self-Compassion Scale Short Form. WEMWBS: Warwick-Edinburgh Mental Well-Being Scale. PSS: Perceived Stress Scale. PHQ-9: Patient Health Questionnaire-9. GAD-7: General Anxiety Disorder-7. MBI-ES: Maslach Burnout Inventory-Educators Survey. OR: odds ratio. 95% CI: 95% confidence interval.

Supplementary material S9: Raw descriptive data by arm

|  |  | Self-taught (*n* = 80) |  | Instructor-led (*n* = 86) |
| --- | --- | --- | --- | --- |
| Variable | Time | Mean (SD) |  | Mean (SD) |
| FFMQ-SF | T0 | 51.44 (6.56) |  | 51.59 (7.01) |
|  | T1 | 52.39 (7.10) |  | 55.27 (6.78) |
| SCS-SF | T0 | 3.21 (0.85) |  | 3.21 (0.77) |
|  | T1 | 3.41 (0.76) |  | 3.74 (0.66) |
| WEMWBS | T0 | 49.08 (7.38) |  | 49.04 (7.11) |
|  | T1 | 50.81 (7.79) |  | 53.45 (6.60) |
| PSS | T0 | 15.00 (7.06) |  | 16.34 (7.29) |
|  | T1 | 14.95 (6.73) |  | 12.76 (5.81) |
| PHQ-9 | T0 | 4.58 (3.84) |  | 4.99 (3.50) |
|  | T1 | 4.60 (4.39) |  | 3.34 (3.15) |
| GAD-7 | T0 | 3.84 (4.37) |  | 4.74 (4.33) |
|  | T1 | 4.04 (4.14) |  | 3.27 (3.26) |
| MBI-ES | T0 | 36.55 (18.49) |  | 40.27 (16.34) |
|  | T1 | 35.73 (18.63) |  | 36.38 (18.31) |

Descriptive data are raw means and SDs. FFMQ-SF: Five Facets Mindfulness Questionnaire Short Form. SCS-SF: Self-Compassion Scale Short Form (excluding the “mindfulness” facet). WEMWBS: Warwick-Edinburgh Mental Well-Being Scale. PSS: Perceived Stress Scale. PHQ-0: Patient Health Questionnaire-9. GAD-7: General Anxiety Disorder-7. MBI-ES: Maslach Burnout Inventory-Educators Survey. Self-taught: *k* = 23 schools. Instructor-led: *k* = 18 schools.

Supplementary Material S10: Self-taught and instructor-led within-group sensitivity analysis of mechanisms and outcomes adjusted for teacher’s gender, age, years of teaching experience and the baseline levels

| Group/Variable |  | d | B | (95% CI) | p |
| --- | --- | --- | --- | --- | --- |
| Self-taught (n = 80) |  |  |  |  |  |
| FFMQ-SF |  | 0.26 | 0.96 | (-0.43 to 2.35) | .176 |
| SCS-SF |  | 0.49 | 0.18 | (0.03 to 0.34) | .022 |
| WEMWBS |  | 0.37 | 1.68 | (0.08 to 3.27) | .039 |
| PSS |  | 0.01 | 0.00 | (-1.66 to 1.67) | .996 |
| PHQ-9 |  | 0.01 | 0.02 | (-1.42 to 1.45) | .981 |
| GAD-7 |  | 0.08 | 0.25 | (-0.75 to 1.25) | .622 |
| MBI-ES |  | -0.09 | -0.62 | (-3.66 to 2.41) | .687 |
| Instructor-led (n = 86) |  |  |  |  |  |
| FFMQ-SF |  | 0.91 | 3.78 | (2.44 to 5.11) | <.001 |
| SCS-SF |  | 1.05 | 0.58 | (0.41 to 0.75) | <.001 |
| WEMWBS |  | 1.25 | 4.48 | (3.30 to 5.65) | <.001 |
| PSS |  | -0.88 | -3.57 | (-4.91 to -2.24) | <.001 |
| PHQ-9 |  | -0.73 | -1.65 | (-2.39 to -0.92) | <.001 |
| GAD-7 |  | -0.74 | -1.53 | (-2.22 to -0.85) | <.001 |
| MBI-ES |  | -0.48 | -3.97 | (-6.97 to -0.96) | .010 |

FFMQ-SF: Five Facets Mindfulness Questionnaire Short Form. SCS-SF: Self-Compassion Scale Short Form. WEMWBS: Warwick-Edinburgh Mental Well-Being Scale. PSS: Perceived Stress Scale. PHQ-9: Patient Health Questionnaire-9. GAD-7: General Anxiety Disorder-7. MBI-ES: Maslach Burnout Inventory-Educators Survey. d: Cohen’s d effect size using adjusted marginal means and SDs. B: unstandardized regression coefficient (95% Confidence Interval). Adjusted models were carried out using hierarchical mixed linear regressions with subjects and schools (clusters) as random effects, controlling for teacher’s gender, age, years of teaching experience and the baseline level of the corresponding variable. Self-taught: k = 23 schools. Instructor-led: k = 18 schools.

Supplementary Material S11: Self-taught and instructor-led within-group analysis adjusted for expectancy, reading the book and weekly days of practice for the sensitivity analysis of mechanisms and outcomes

| Group/Variable | d | B | (95% CI) | p |
| --- | --- | --- | --- | --- |
| Self-taught (n = 80) |  |  |  |  |
| FFMQ-SF | 0.14 | 1.14 | (-0.40 to 2.68) | .148 |
| SCS-SF | 0.20 | 0.18 | (0.01 to 0.35) | .040 |
| WEMWBS | 0.24 | 1.91 | (0.11 to 3.71) | .038 |
| PSS | -0.01 | -0.01 | (-1.88 to 1.88) | .998 |
| PHQ-9 | 0.06 | 0.40 | (-1.28 to 2.08) | .641 |
| GAD-7 | 0.05 | 0.29 | (-0.91 to 1.49) | .632 |
| MBI-ES | -0.02 | -0.22 | (-3.73 to 3.30) | .904 |
| Instructor-led (n = 86) |  |  |  |  |
| FFMQ-SF | 0.48 | 3.65 | (2.25 to 5.05) | <.001 |
| SCS-SF | 0.72 | 0.58 | (0.41 to 0.75) | <.001 |
| WEMWBS | 0.67 | 4.53 | (2.90 to 6.16) | <.001 |
| PSS | -0.57 | -3.50 | (-5.10 to -1.90) | <.001 |
| PHQ-9 | -0.41 | -1.64 | (-2.51 to -0.77) | <.001 |
| GAD-7 | -0.45 | -1.49 | (-2.03 to -0.94) | <.001 |
| MBI-ES | -0.23 | -3.72 | (-7.43 to 0.00) | .048 |

FFMQ-SF: Five Facets Mindfulness Questionnaire Short Form. SCS-SF: Self-Compassion Scale Short Form. WEMWBS: Warwick-Edinburgh Mental Well-Being Scale. PSS: Perceived Stress Scale. PHQ-9: Patient Health Questionnaire-9. GAD-7: General Anxiety Disorder-7. MBI-ES: Maslach Burnout Inventory-Educators Survey. d: Cohen’s d effect size using adjusted marginal means and SDs. B: unstandardized regression coefficient (95% Confidence Interval). Adjusted models were carried out using hierarchical mixed linear regressions with subjects and schools (clusters) as random effects, controlling for expectancy, reading the book and weekly days of practice. Self-taught: k = 23 schools. Instructor-led: k = 18 schools.

Supplementary Material S12: Self-taught and instructor-led within-group analysis with imputed values for the sensitivity analysis of mechanisms and outcomes

| Group/Variable |  | d | B | (95% CI) | p |
| --- | --- | --- | --- | --- | --- |
| Self-taught (n = 101) |  |  |  |  |  |
| FFMQ-SF |  | 0.33 | 2.45 | (0.47 to 4.42) | .015 |
| SCS-SF |  | 0.27 | 0.21 | (0.12 to 0.30) | <.001 |
| WEMWBS |  | 0.34 | 2.27 | (0.65 to 3.90) | .006 |
| PSS |  | -0.02 | -0.14 | (-1.61 to 1.32) | .850 |
| PHQ |  | -0.09 | -0.61 | (-1.41 to 0.19) | .134 |
| GAD |  | -0.03 | -0.11 | (-0.83 to 0.60) | .756 |
| MBI |  | 0.01 | 0.13 | (-2.44 to 2.70) | .922 |
| Instructor-led (n = 105) |  |  |  |  |  |
| FFMQ-SF |  | 0.57 | 4.70 | (2.03 to 7.38) | .001 |
| SCS-SF |  | 0.60 | 0.58 | (0.29 to 0.87) | <.001 |
| WEMWBS |  | 0.55 | 5.84 | (2.13 to 9.55) | .002 |
| PSS |  | -0.51 | -4.61 | (-7.58 to -1.64) | .002 |
| PHQ |  | -0.32 | -3.12 | (-6.59 to 0.36) | .079 |
| GAD |  | -0.13 | -3.66 | (-8.65 to 1.33) | .150 |
| MBI |  | -0.15 | -2.88 | (-8.78 to 3.02) | .339 |

FFMQ-SF: Five Facets Mindfulness Questionnaire Short Form. SCS-SF: Self-Compassion Scale Short Form. WEMWBS: Warwick-Edinburgh Mental Well-Being Scale. PSS: Perceived Stress Scale. PHQ-9: Patient Health Questionnaire-9. GAD-7: General Anxiety Disorder-7. MBI-ES: Maslach Burnout Inventory-Educators Survey. B: unstandardized regression coefficient (95% Confidence Interval). Adjusted models were carried out using hierarchical mixed linear regressions with subjects and schools (clusters) as random effects. Imputations were calculated from chained equations using linear regressions that included the variables introduced in the analyses, pre-intervention covariates included in the adjusted models, the cluster size at baseline, and those baseline variables that were significantly related to non-response (i.e. FFMQ-SF, SCS-SF, WEMWBS, PSS, PHQ-9, GAD-7, MBI-ES, expectancy, cluster size at baseline, age, gender, number of years teaching, percentage of free school meals, group). Analysis including the total sample (i.e. 43 schools with 206 teachers).

Supplementary Material S13: Adjusted models controlling for teacher’s gender, age, years of teaching experience and the baseline levels for the between-group sensitivity analysis of mechanisms and outcomes

|  |  | Self-taught (n = 80) | Instructor-led (n = 86) |  | | |  |
| --- | --- | --- | --- | --- | --- | --- | --- |
| Variable | Time | Mean (SD) | Mean (SD) | d | B | (95% CI) | p |
| FFMQ-SF ^†^ | T0 | 51.37 (1.40) | 51.38 (1.08) |  |  |  |  |
|  | T1 | 52.33 (6.40) | 55.15 (5.60) | 0.46 | 2.82 | (0.90 to 4.74) | .004 |
| SCS-SF ^†^ | T0 | 3.06 (0.23) | 3.04 (0.15) |  |  |  |  |
|  | T1 | 3.24 (0.64) | 3.62 (0.71) | 0.67 | 0.40 | (0.17 to 0.63) | .001 |
| WEMWBS ^†^ | T0 | 49.10 (1.28) | 49.04 (1.10) |  |  |  |  |
|  | T1 | 50.77 (6.81) | 53.52 (5.10) | 0.51 | 2.80 | (0.83 to 4.77) | .005 |
| PSS ^† ‡^ | T0 | 15.47 (1.64) | 15.75 (1.47) |  |  |  |  |
|  | T1 | 15.48 (7.19) | 12.17 (5.48) | -0.70 | -3.58 | (-5.70 to -1.46) | .001 |
| PHQ-9 ^†^ | T0 | 4.72 (1.02) | 4.77 (0.89) |  |  |  |  |
|  | T1 | 4.74 (6.17) | 3.12 (2.97) | -0.39 | -1.67 | (-3.29 to -0.05) | .043 |
| GAD-7 ^†^ | T0 | 4.14 (1.01) | 4.41 (0.92) |  |  |  |  |
|  | T1 | 4.39 (4.56) | 2.88 (2.65) | -0.69 | -1.78 | (-3.00 to -0.57) | .004 |
| MBI-ES ^†^ | T0 | 37.80 (2.68) | 38.38 (2.09) |  |  |  |  |
|  | T1 | 37.17 (13.24) | 34.42 (13.80) | -0.45 | -3.34 | (-7.60 to 0.92) | .124 |

FFMQ-SF: Five Facets Mindfulness Questionnaire Short Form. SCS-SF: Self-Compassion Scale Short Form. WEMWBS: Warwick-Edinburgh Mental Well-Being Scale. PSS: Perceived Stress Scale. PHQ-9: Patient Health Questionnaire-9. GAD-7: General Anxiety Disorder-7. MBI-ES: Maslach Burnout Inventory-Educators Survey. Descriptive are adjusted marginal means and SDs. d: Cohen’s d effect size using adjusted marginal means and SDs. B: unstandardized regression coefficient (95% Confidence Interval). Adjusted models were carried out using hierarchical mixed linear regressions with subjects and schools (clusters) as random effects, controlling for teacher’s gender, age, years of teaching experience and the baseline level of the corresponding variable. ^†^ The baseline level was a significant positively related covariate (p < .001). ^‡^ Years of teaching was a significant covariate (B = -0.06, p = .032). Complete cases analysis (self-taught: k = 23 schools; instructor-led: k = 18 schools).

Supplementary Material S14: Adjusted models controlling for expectancy, weekly days of mindfulness meditation practice, and reading the book for the between-group sensitivity analysis of mechanisms and outcomes

|  |  | Self-taught (n = 80) | Instructor-led (n = 86) |  | | |  |
| --- | --- | --- | --- | --- | --- | --- | --- |
| Variable | Time | Mean (SD) | Mean (SD) | d | B | (95% CI) | p |
| FFMQ-SF ^a, c^ | T0 | 52.11 (9.92) | 50.91 (8.43) |  |  |  |  |
|  | T1 | 53.25 (10.37) | 54.56 (6.12) | 0.28 | 2.51 | (0.43 to 4.59) | .018 |
| SCS-SF ^b, c^ | T0 | 3.19 (1.16) | 3.03 (0.83) |  |  |  |  |
|  | T1 | 3.36 (1.07) | 3.61 (0.65) | 0.40 | 0.40 | (0.16 to 0.64) | .001 |
| WEMWBS ^c^ | T0 | 49.41 (10.01) | 48.36 (8.44) |  |  |  |  |
|  | T1 | 51.31 (7.15) | 52.89 (5.19) | 0.28 | 2.62 | (0.20 to 5.05) | .034 |
| PSS ^d^ | T0 | 14.68 (9.03) | 16.32 (7.32) |  |  |  |  |
|  | T1 | 14.67 (8.49) | 12.82 (4.63) | -0.42 | -3.50 | (-5.96 to -1.04) | .005 |
| PHQ-9 ^d^ | T0 | 4.25 (5.28) | 5.06 (4.08) |  |  |  |  |
|  | T1 | 4.65 (6.52) | 3.42 (3.71) | -0.43 | -2.04 | (-3.93 to -0.15) | .039 |
| GAD-7 ^c^ | T0 | 3.74 (5.18) | 4.78 (3.34) |  |  |  |  |
|  | T1 | 4.03 (5.46) | 3.29 (3.06) | -0.41 | -1.78 | (-3.09 to -0.46) | .008 |
| MBI-ES | T0 | 34.74 (25.85) | 43.08 (14.56) |  |  |  |  |
|  | T1 | 34.52 (24.06) | 39.37 (23.55) | -0.17 | -3.50 | (-8.61 to 1.62) | .180 |

FFMQ-SF: Five Facets Mindfulness Questionnaire Short Form. SCS-SF: Self-Compassion Scale Short Form. WEMWBS: Warwick-Edinburgh Mental Well-Being Scale. PSS: Perceived Stress Scale. PHQ-9: Patient Health Questionnaire-9. GAD-7: General Anxiety Disorder-7. MBI-ES: Maslach Burnout Inventory-Educators Survey. Descriptive are adjusted marginal means and SDs. d: Cohen’s d effect size using adjusted marginal means and SDs. B: unstandardized regression coefficient (95% Confidence Interval). Adjusted models were carried out using hierarchical mixed linear regressions with subjects and schools (clusters) as random effects, controlling for expectancy, reading the book and weekly days of practice. Expectancy was a significant covariate in ^a^ (p < .001) and ^b^ (p < .05). Weekly days of mindfulness practice was a significant covariate in ^c^ (p < .001) and ^d^ (p < .01). Significant covariates showed the same valence than the corresponding slope for the group-by-wave interaction. Complete cases analysis (self-taught: k = 23 schools; instructor-led: k = 18 schools).

Supplementary Material S15: Imputed between-group sensitivity analysis of mechanisms and outcomes

|  |  | Self-taught (n = 101) | Instructor-led (n = 105) |  | | | |
| --- | --- | --- | --- | --- | --- | --- | --- |
| Variable | Time | Mean (SD) | Mean (SD) | d | B | (95% CI) | p |
| FFMQ-SF | T0 | 50.98 (9.35) | 50.68 (9.27) |  |  |  |  |
|  | T1 | 53.42 (9.05) | 55.38 (6.38) | 0.24 | 2.26 | (-1.07 to 5.59) | .183 |
| SCS-SF | T0 | 3.09 (1.01) | 2.99 (1.03) |  |  |  |  |
|  | T1 | 3.30 (1.11) | 3.57 (0.72) | 0.36 | 0.37 | (0.07 to 0.67) | .017 |
| WEMWBS | T0 | 49.26 (7.24) | 48.17 (10.66) |  |  |  |  |
|  | T1 | 51.53 (9.25) | 54.01 (11.17) | 0.39 | 3.57 | (-0.48 to 7.62) | .084 |
| PSS | T0 | 14.78 (7.54) | 16.75 (8.40) |  |  |  |  |
|  | T1 | 14.64 (8.64) | 12.14 (9.43) | -0.52 | -4.47 | (-7.78 to -1.16) | .008 |
| PHQ-9 | T0 | 4.63 (6.74) | 5.57 (9.94) |  |  |  |  |
|  | T1 | 4.01 (7.11) | 2.45 (9.22) | -0.29 | -2.50 | (-6.07 to 1.06) | .168 |
| GAD-7 | T0 | 3.67 (4.42) | 5.81 (11.78) |  |  |  |  |
|  | T1 | 3.56 (4.52) | 2.15 (14.96) | -0.40 | -3.55 | (-8.59 to 1.49) | .169 |
| MBI-ES | T0 | 33.90 (19.60) | 40.69 (22.95) |  |  |  |  |
|  | T1 | 34.02 (22.81) | 37.81 (21.42) | -0.14 | -3.01 | (-9.44 to 3.42) | .359 |

FFMQ-SF: Five Facets Mindfulness Questionnaire Short Form. SCS-SF: Self-Compassion Scale Short Form. WEMWBS: Warwick-Edinburgh Mental Well-Being Scale. PSS: Perceived Stress Scale. PHQ-9: Patient Health Questionnaire-9. GAD-7: General Anxiety Disorder-7. MBI-ES: Maslach Burnout Inventory-Educators Survey. Descriptive are adjusted marginal means and SDs. d: Cohen’s d effect size using adjusted marginal means and SDs. B: unstandardized regression coefficient (95% Confidence Interval) from hierarchical mixed linear regressions with subjects and schools (clusters) as random effects. Imputations were calculated from chained equations using linear regressions that included the variables introduced in the analyses, pre-intervention covariates included in the adjusted models, the cluster size at baseline, and those baseline variables that were significantly related to non-response (i.e. FFMQ-SF, SCS-SF, WEMWBS, PSS, PHQ-9, GAD-7, MBI-ES, expectancy, cluster size at baseline, age, gender, number of years teaching, percentage of free school meals, group). Analysis including the total sample (i.e. 43 schools with 206 teachers).

Supplementary Material S16: Implementation variables as predictors of outcome improvements in the self-taught and instructor-led ways of delivering the mindfulness program

|  | Self-taught (n = 80) | |  | Instructor-led (n = 86) | |
| --- | --- | --- | --- | --- | --- |
| IV/DV | B (95% CI) | p |  | B (95% CI) | p |
| Expectancy |  |  |  |  |  |
| FFMQ | 1.24 (0.62 to 1.86) | < .001 |  | 1.60 (0.93 to 2.27) | < .001 |
| SCS | 0.13 (0.07 to 0.19) | < .001 |  | 0.08 (-0.01 to 0.17) | .093 |
| WEMWBS | 0.57 (0.01 to 1.12) | .045 |  | 1.19 (0.47 to 1.91) | .001 |
| PSS | -0.41 (-1.00 to 0.18) | .176 |  | -1.20 (-1.84 to -0.57) | < .001 |
| PHQ9 | 0.05 (-0.41 to 0.51) | .839 |  | -0.74 (-1.03 to -0.45) | < .001 |
| GAD | 0.05 (-0.44 to 0.53) | .849 |  | -0.34 (-0.72 to 0.05) | .086 |
| MBI | -0.49 (-1.85 to 0.86) | .476 |  | 0.12 (-1.55 to 1.79) | .892 |
| Practice |  |  |  |  |  |
| FFMQ | 2.05 (1.00 to 3.09) | < .001 |  | 1.64 (0.82 to 2.46) | < .001 |
| SCS | 0.30 (0.20 to 0.40) | < .001 |  | 0.20 (0.14 to 0.27) | < .001 |
| WEMWBS | 2.30 (1.27 to 3.33) | < .001 |  | 0.99 (0.30 to 1.68) | .005 |
| PSS | -1.54 (-2.40 to -0.69) | < .001 |  | -0.98 (-1.85 to -0.12) | .025 |
| PHQ-9 | -1.12 (-1.68 to -0.55) | < .001 |  | -0.37 (-0.69 to -0.04) | .026 |
| GAD-7 | -0.93 (-1.45 to -0.42) | < .001 |  | -0.72 (-1.15 to -0.29) | .001 |
| MBI-ES | -0.19 (-2.16 to 1.78) | .849 |  | -1.08 (-3.57 to 1.41) | .396 |
| Book |  |  |  |  |  |
| FFMQ | 1.37 (-0.21 to 2.94) | .089 |  | 0.26 (-1.35 to 1.87) | .750 |
| SCS | 0.22 (0.01 to 0.43) | .043 |  | 0.14 (-0.03 to 0.30) | .100 |
| WEMWBS | 1.87 (0.52 to 3.22) | .007 |  | 0.09 (-1.67 to 1.84) | .922 |
| PSS | -1.35 (-2.60 to -0.10) | .034 |  | 0.42 (-1.40 to 2.23) | .653 |
| PHQ-9 | -1.02 (-1.67 to -0.37) | .002 |  | 0.26 (-0.45 to 0.98) | .473 |
| GAD-7 | -0.70 (-1.43 to 0.03) | .058 |  | 0.15 (-0.48 to 0.79) | .636 |
| MBI-ES | -2.86 (-5.91 to 0.19) | .066 |  | -0.32 (-2.68 to 2.04) | .791 |

Expectancy: level of expectancy at the second week of the program. Practice: weekly days of mindfulness meditation practice. Book: reading the book. FFMQ-SF: Five Facets Mindfulness Questionnaire Short Form. SCS-SF: Self-Compassion Scale Short Form. WEMWBS: Warwick-Edinburgh Mental Well-Being Scale. PSS: Perceived Stress Scale. PHQ-9: Patient Health Questionnaire-9. GAD-7: General Anxiety Disorder-7. MBI-ES: Maslach Burnout Inventory-Educators Survey. B: unstandardized regression coefficient using hierarchical linear mixed models with subjects and schools as random effects. Complete cases analysis (self-taught: k = 23 schools; instructor-led: k = 18 schools).

Supplementary Material S17: Path estimates and indirect effects of frequency of mindfulness meditation practice through FFMQ-SF (i) on teacher outcomes

| Group/DV | R^2^ | aw (SE) | p | bw (SE) | p | cw’ (SE) | p | IEs (MC 95% CI) |
| --- | --- | --- | --- | --- | --- | --- | --- | --- |
| Self-taught  (n = 80) |  |  |  |  |  |  |  |  |
| WEMWBS | .20 | 0.83 (0.36) | .022 | 0.51 (0.17) | .003 | 0.47 (0.53) | .375 | 0.43 ( 0.06 to 0.90) |
| PSS | .15 | 0.83 (0.36) | .022 | -0.50 (0.20) | .015 | -0.32 (0.41) | .443 | -0.41 (-0.99 to -0.03) |
| PHQ-9 | .11 | 0.83 (0.36) | .022 | -0.27 (0.11) | .022 | -0.15 (0.25) | .555 | -0.22 (-0.52 to -0.02) |
| GAD-7 | .17 | 0.83 (0.36) | .022 | -0.33 (0.13) | .013 | -0.13 (0.35) | .697 | -0.28 (-0.60 to -0.04) |
| MBI-ES | .14 | 0.83 (0.36) | .022 | -0.92 (0.31) | .003 | -1.09 (0.65) | .097 | -0.76 (-1.72 to -0.09) |
|  |  |  |  |  |  |  |  |  |
| Instructor-led  (n = 86) |  |  |  |  |  |  |  |  |
| WEMWBS | .26 | 1.54 (0.57) | .007 | 0.46 (0.08) | <.001 | 0.23 (0.39) | .562 | 0.71 ( 0.18 to 1.38) |
| PSS | .14 | 1.54 (0.57) | .007 | -0.31 (0.07) | <.001 | -0.42 (0.54) | .439 | -0.48 (-1.02 to -0.09) |
| PHQ-9 | .23 | 1.54 (0.57) | .007 | -0.22 (0.04) | <.001 | -0.20 (0.19) | .303 | -0.34 (-0.62 to -0.09) |
| GAD-7 | .08 | 1.54 (0.57) | .007 | -0.13 (0.05) | .012 | -0.11 (0.19) | .591 | -0.20 (-0.44 to -0.04) |
| MBI-ES | .29 | 1.54 (0.57) | .007 | -1.04 (0.25) | <.001 | -0.75 (1.00) | .457 | -1.61 (-3.18 to -0.34) |

DV: dependent variable. aw: unstandardized estimated of path “aw” in the mediating model. bw: unstandardized estimated of path “bw” in the mediating model. cw’: unstandardized direct effects after controlling for the indirect effects. SE: standard error. IEs: indirect effects (95% confidence interval for the indirect effect based on a Monte Carlo simulation of the joint distribution of the corresponding slopes using 20,000 random draws from the parameter estimates and their associated asymptotic variances and covariance). FFMQ-SF: Five Facet Mindfulness Questionnaire Short Form. WEMWBS: Warwick-Edinburgh Mental Well-Being Scale. PSS: Perceived Stress Scale. PHQ-9: Patient Health Questionnaire-9. GAD-7: General Anxiety Disorder-7. MBI-ES: Maslach Burnout Inventory-Educators Survey. Complete cases analysis (self-taught: k = 23 schools; instructor-led: k = 18 schools).

Supplementary Material S18: Path estimates and indirect effects of frequency of mindfulness meditation practice through SCS-SF (ii) on teacher outcomes

| Group/DV | R^2^ | aw (SE) | p | bw (SE) | p | cw’ (SE) | p | IEs (MC 95% CI) |
| --- | --- | --- | --- | --- | --- | --- | --- | --- |
| Self-taught  (n = 80) |  |  |  |  |  |  |  |  |
| WEMWBS | .16 | 0.07 (0.04) | .091 | 3.76 (1.82) | .039 | 0.64 (0.55) | .245 | 0.26 (-0.07 to 0.82) |
| PSS | .14 | 0.07 (0.04) | .091 | -4.13 (1.84) | .025 | -0.19 (0.44) | .677 | -0.28 (-0.85 to 0.08) |
| PHQ-9 | .16 | 0.07 (0.04) | .091 | -2.77 (1.21) | .022 | -0.12 (0.24) | .633 | -0.19 (-0.57 to 0.05) |
| GAD-7 | .18 | 0.07 (0.04) | .091 | -2.88 (1.06) | .006 | -0.06 (0.31) | .851 | -0.20 (-0.56 to -0.05) |
| MBI-ES | .17 | 0.07 (0.04) | .091 | -8.68 (3.09) | .005 | -0.93 (0.66) | .163 | -0.60 (-1.65 to 0.14) |
|  |  |  |  |  |  |  |  |  |
| Instructor-led  (n = 86) |  |  |  |  |  |  |  |  |
| WEMWBS | .29 | 0.13 (0.08) | .109 | 4.10 (0.54) | <.001 | 0.41 (0.34) | .229 | 0.52 (-3.81 to 4.87) |
| PSS | .18 | 0.13 (0.08) | .109 | -3.15 (0.90) | <.001 | -0.50 (0.46) | .277 | -0.40 (-1.03 to 0.06) |
| PHQ-9 | .16 | 0.13 (0.08) | .109 | -1.55 (0.27) | <.001 | -0.05 (0.18) | .764 | -0.20 (-0.47 to 0.03) |
| GAD-7 | .05 | 0.13 (0.08) | .109 | -0.70 (0.34) | .038 | -0.22 (0.17) | .196 | -0.09 (-0.27 to 0.02) |
| MBI-ES | .29 | 0.13 (0.08) | .109 | -8.92 (1.77) | <.001 | -1.23 (1.00) | .220 | -1.13 (-2.75 to 0.20) |

DV: dependent variable. aw: unstandardized estimated of path “aw” in the mediating model. bw: unstandardized estimated of path “bw” in the mediating model. cw’: unstandardized direct effects after controlling for the indirect effects. SE: standard error. IEs: indirect effects (95% confidence interval for the indirect effect based on a Monte Carlo simulation of the joint distribution of the corresponding slopes using 20,000 random draws from the parameter estimates and their associated asymptotic variances and covariance). SCS-SF: Self-Compassion Scale Short Form. WEMWBS: Warwick-Edinburgh Mental Well-Being Scale. PSS: Perceived Stress Scale. PHQ-9: Patient Health Questionnaire-9. GAD-7: General Anxiety Disorder-7. MBI-ES: Maslach Burnout Inventory-Educators Survey. Complete cases analysis (self-taught: k = 23 schools; instructor-led: k = 18 schools).
